# Slice Level Classification of Parathyroid Adenoma Using Arterial Phase CT Images with Hybrid Light Attention Mechanism Based Residual Framework

**DOI:** 10.3390/bioengineering13080850

**Published:** 2026-07-23

**Authors:** Muhammad Saad Bin Abdul Ghaffar, Radhwan A. A. Saleh, Humam AbuAlkebash, Zead Saleh, Muhammed Kızıltepe, Burcu Alparslan, Huseyin Metin Ertunc

**Affiliations:** 1 Department of Mechatronics Engineering, Kocaeli University, Kocaeli 41001, Türkiye; saad.ghaffar@kocaeli.edu.tr (M.S.B.A.G.); hmertunc@kocaeli.edu.tr (H.M.E.); 2 Interdisciplinary Research Center for Construction and Building Materials, King Fahd University of Petroleum & Minerals (KFUPM), Dhahran 31261, Saudi Arabia; 3 Robotic and Artificial Intelligence Program, Ford Otosan İhsaniye Automotive MYO, Kocaeli University, Kocaeli 41650, Türkiye; habualkebash@kocaeli.edu.tr; 4 Department of Industrial Engineering, College of Engineering, University of Business and Technology, Jeddah 21361, Saudi Arabia; z.saleh@ubt.edu.sa; 5 Department of Radiology, Kocaeli University, Kocaeli 41001, Türkiye; muhammed.kiziltepe@kocaeli.edu.tr (M.K.); burcu.alparslan@kocaeli.edu.tr (B.A.)

**Keywords:** medical imaging, deep learning, attention mechanisms, clinical decision support, computed tomography, parathyroid adenoma, diagnostic imaging, oncology, explainable AI (XAI)

## Abstract

Artificial intelligence (AI) is transforming oncologic imaging by enabling automated, accurate, and scalable diagnostic decision support. However, successful clinical translation requires not only strong predictive performance but also interpretability, transparency, and clinician confidence. We present an attention-guided deep learning framework for automated slice-level classification of parathyroid adenoma (PTA) from arterial-phase computed tomography (CT) images. The framework incorporates lightweight hierarchical attention mechanisms within residual neural networks to enhance feature representation and contextual understanding while maintaining computational efficiency for real-world deployment. Three novel architectures were developed: the Residual Block Light Attention Network (Res-BLANet), Residual Stage Light Attention Network (Res-SLANet), and Residual Layer Light Attention Network (Res-LLANet). Models were trained and evaluated on a rigorously curated, expert-annotated dataset of 63 patients, including 39 pathologically confirmed PTA cases, with 350–450 slices per patient. Training employed Hounsfield unit normalization, extensive data augmentation, and 10-fold cross-validation to ensure robust performance assessment. A key innovation is the integration of hierarchical attention modules that generate attention maps at multiple network levels, enabling qualitative visualization of diagnostically relevant regions and providing exploratory insight into the model’s decision-making process. Experimental evaluation showed strong diagnostic performance. Res-SLANet achieved the highest overall accuracy (87.37%), precision (92.56%), and F1-score (86.69%), while Res-LLANet attained the highest sensitivity (96.0%) on independent testing. These results should be interpreted as preliminary, given the limited size of the independent patient-level test cohort (n = 3). Res-BLANet delivered substantially faster inference with minimal computational overhead. These findings demonstrate that hierarchical attention-guided deep learning can achieve accurate and computationally efficient PTA detection from CT imaging while providing qualitative visual insights into the model’s decision-making process. The proposed framework represents a promising approach toward more interpretable AI-assisted diagnostic systems for oncologic imaging.

## 1. Introduction

**Figure 1:**
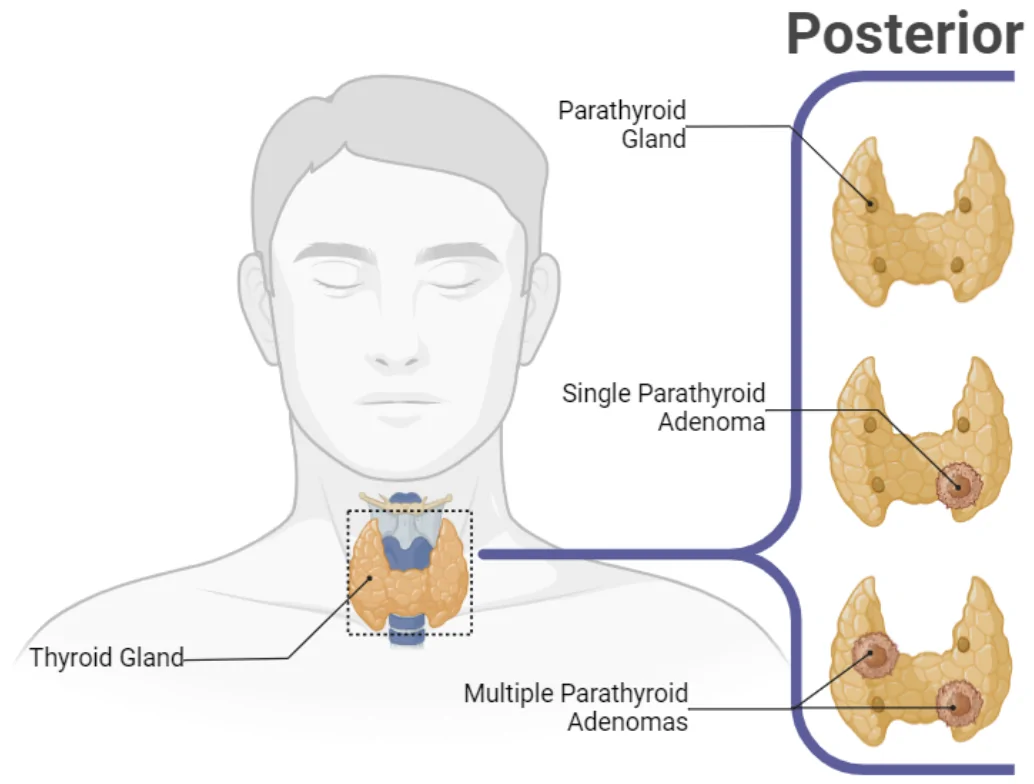
Anatomy of Parathyroid Adenoma.

Calcium regulation is an essential process of the human body, which governs critical processes such as bone metabolism, muscle contraction, and nerve signal transmission [1]. The parathyroid glands, which normally comprise four small structures located on the posterior side of the thyroid gland as shown in (Figure 1), are the primary calcium regulators, which balance it through the secretion of parathyroid hormone (PTH) [2,3]. The most common pathology disrupting this system is the development of a parathyroid adenoma (PTA), a noncancerous growth of parathyroid gland that leads to the overproduction of PTH. This condition, known as primary hyperparathyroidism (PHPT), is most frequently caused by a single adenoma (single-gland disease, SGD), but can also arise from multiple affected glands (multi-gland disease, MGD) [1], as illustrated in Figure 1. While PHPT is often detected incidentally through routine blood tests showing elevated calcium levels, symptomatic patients may present with significant clinical sequelae, including nephrolithiasis, bone pain, osteoporosis, and neuropsychiatric disturbances [4]. The successful treatment of PHPT hinges on the definitive surgical removal of the hypersecreting adenomatous tissue. Historically, the standard procedure was a bilateral neck exploration (BNE), wherein the surgeon examined and evaluated each of the four parathyroid glands by looking at both sides of the neck. In order to ensure that no other abnormal glands were missed, this method sought to locate and remove the swollen (adenomatous) gland or glands. Despite being successful, it was a more complex and intrusive procedure that required more time for rehabilitation and surgery than more recent specialized methods. However, innovations have improved this approach in medical imaging, shifting the paradigm towards a targeted, minimally invasive parathyroidectomy (MIP) [5]. The effectiveness of MIP is directly linked to the accurate preoperative localization of the culprit adenoma(s). Precise localization enables the surgeon to do a confined dissection through a smaller incision. This significantly benefits the patients in terms of clinical setting such as reduced operative time, less postoperative pain, shorter hospital stays, and a lower risk of complications like hyperparathyroidism or damage to the recurrent laryngeal nerve [5]. As a result, the financial burden on both the patient and the diagnostic imaging workflow is substantially reduced. On the other hand, if the radiologist is unable to detect the adenoma accurately, it’s possible that surgery might fail, which may result in recurring or permanent hyperparathyroidism, which in return would increase the difficulty for re-surgery and patient morbidity [6]. Therefore, robust and reliable preoperative diagnosis is not merely a task but a critical prerequisite for optimal surgical planning and successful patient outcomes.

Nowadays, a wide range of imaging modalities is available for the preoperative localization of parathyroid adenomas, each offering a unique profile in terms of advantages and limitations [7]. Initial diagnosis typically begins with cervical ultrasound (US), a widely accessible, cost-effective, and radiation-free technique valued for its real-time capabilities [8,9]. Another primary modality is 99mTc-sestamibi scintigraphy, a nuclear medicine study that leverages the preferential uptake of a radiotracer by the mitochondria-rich oxyphil cells abundant in PTAs [10]. To automate the adenoma detection, the diagnostic performance of scintigraphy has been notably enhanced by the integration of artificial intelligence [11], with the help of deep learning models [12,13]. Advanced imaging modalities are employed for cases that are more complicated and unclear. Magnetic Resonance Imaging (MRI) offers superior soft-tissue contrast without ionizing radiation, making it a vital tool, especially for the ectopic or deep-seated adenomas [14]. Its diagnostic effectiveness is even further improved with the use of advanced sequencing and contrast agents [15]. Similarly, Positron Emission Tomography (PET), which typically utilizes the tracer 18F-fluorocholine, has emerged as a highly sensitive modality that often outperforms the traditional imaging modalities [16], and is typically reserved for extreme cases such as reoperative surgeries or cases where the detection from other modalities has failed [17,18]. Among these cutting-edge techniques, Four-Dimensional Computed Tomography (4D-CT) has emerged as a key technology because of its high spatial resolution and its ability to capture dynamic tissue perfusion [19]. The 4D-CT involves capturing the images across multiple contrast-enhanced phases to record the rapid “wash-in” and subsequent “wash-out” of contrast agent in a hypervascular adenoma. This multiphasic imaging is essential for differentiating PTAs from adjacent structures like the thyroid gland and lymph nodes [20], making it as an extremely sensitive tool, particularly for localizing ectopic adenomas [21,22].

**Figure 2:**
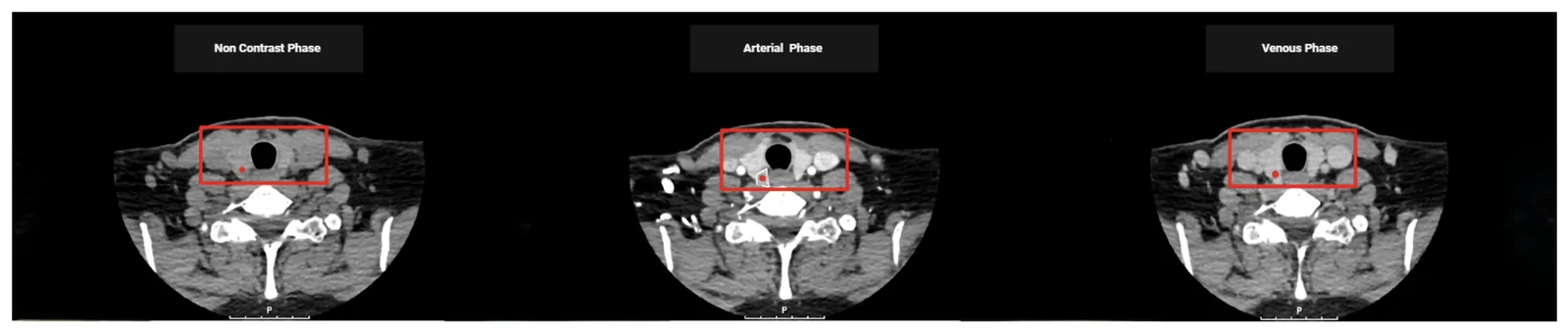
Difference in the contrast of 3 phases of 4D-CT images taken for the diagnosis of parathyroid adenoma (Red Dot shows the parathyroid adenoma in these images).

Despite this multiphasic approach being the key to 4D-CT’s diagnostic potential, this technique also introduces significant challenges for both clinicians and patients. The effectiveness of the protocol relies on a thorough analysis across its distinct phases. Prior to the influence of contrast media, the initial non-contrast phase provides a crucial anatomical baseline for the identification of tissue densities [21]. The most critical phase for adenoma identification is the arterial phase, followed by the non-contrast phase, typically 25–30 s post-injection. Due to their hypervascular nature, parathyroid adenomas demonstrate greater enhancement than the surrounding tissues during the arterial phase following contrast administration, making this phase crucial for accurate localization [23]. Subsequently, the rapid “wash-out” of contrast media from the adenoma in the venous phase helps differentiate it from the thyroid gland, which exhibits relatively slower and more prolonged enhancement. In some ambiguous cases where the washout pattern is unclear, an optional delayed phase can further confirm this wash-out pattern [24]. The distinct contrast behaviors across these phases can be visualized in Figure 2. However, this detailed anatomical and functional information comes at a significant cost. The massive volume of image data generated by this multi-phase imaging modality puts a significant cognitive strain on the interpreting radiologist and also on the hospital data storage systems [7]. Accurate differentiation of parathyroid adenomas from normal or other pathological tissues is both time-consuming and inherently subjective because it requires careful comparison of subtle anatomical and contrast variations across hundreds of CT slices. Consequently, diagnostic errors, inter-observer variability, and potential delays resulting from the radiologist’s interpretation may significantly effect the diagnostic accuracy [25]. Furthermore, this procedure presents significant drawbacks for the patient. In comparison with the other modalities like ultrasound or scintigraphy, the repeated scanning through multiple phases results in a substantially higher radiation dose [26], raises concerns about long-term cancer risk, particularly for younger patients or those needing future scans [27,28]. The intravenous iodinated contrast agents, which are a prerequisite, also carry the risk of allergic reactions and contrast-induced nephropathy in patients with pre-existing renal dysfunction [29]. These combined challenges, including high data complexity, interpreter subjectivity, and patient safety concerns, create a critical bottleneck in oncologic and endocrine imaging workflows and highlight the need for more objective, efficient, and automated image analysis approaches.

To address the challenges associated with manual 4D-CT interpretation, recent advances in artificial intelligence and oncologic imaging have increasingly leveraged deep learning frameworks for automated lesion detection and diagnostic decision support [6,30]. To detect the subtle discriminative features of parathyroid adenomas that might be difficult for the human eye to discern, these models can be effectively trained to analyze medical images from a variety of modalities, including 4D-CT, ultrasound, and scintigraphy [25]. Deep learning has the ability to automate the detection by processing the high-dimensional data of 4D-CT scans, offering the potential to significantly improve both diagnostic efficiency and accuracy [31]. However, the practical application of DL for PTA detection faces significant hurdles, including the scarcity of large, annotated datasets and the high computational cost of many advanced models. This leads to a critical research gap that is addressed by creating deep learning solutions that are not only highly accurate but also efficient and practical enough for real-world clinical workflows.

The recent works in medical imaging show that attention-based DL models like Squeeze-and-Excitation (SENet) [32], CBAM [33], Non-local [34], CCNet [35], and Vision Transformers [36] have improved feature representation. Channel-focused modules like SENet ignore spatial dependencies, while CBAM and related hybrid models compress spatial details through global pooling, which limits lesion localization. Non-local and transformer architectures capture long-range dependencies but incur quadratic computational costs, require massive training data, and limited interpretability when applied to volumetric medical scans. Even recent lightweight or probabilistic attention variants [37,38] reduce computation at the expense of fine-grained contextual learning, and current surveys [39] highlight that existing attention frameworks are rarely adapted to 3D or multi-phase medical imaging, such as 4D-CT.

On the other hand, most previous PTA studies have centered on nuclear medicine modalities like 99mTc-sestamibi scintigraphy [10,11,12,13,40], intraoperative autofluorescence [41,42], or PET imaging [43], achieving promising sensitivity yet constrained by single-modality, low-resolution data and weak spatial localization. Hybrid clinical and biochemical models [17,44,45] improved diagnostic reliability but relied on handcrafted feature fusion rather than end-to-end learning. Even the most advanced 4D-CT study using Bi-LSTM networks [19] lacked attention-based spatial modelling and suffered from high false-positive rates and limited interpretability. As a result, a deep learning framework that is efficient, capable of providing qualitative model interpretability, and spatially precise is needed for analyzing CT scans.

This paper introduces a deep learning based residual framework that has been improved with novel hybrid blocks integrated with two novel lightweight, hierarchical attention mechanisms in order to overcome these limitations. These lightweight attention mechanisms integrate with several other components that function at various scales within the network while maintaining the computational efficacy instead of relying on a single, computationally demanding attention module. First, the Block-Level Attention Module (BLAM) suppresses the unimportant background information while highlighting the parathyroid gland’s subtle features and contrast patterns within each small part of the model. The Stage-Level Attention Module (SLAM) establishes connections between the major stages of the network, enabling the aggregation of information from multiple feature levels to capture both fine-grained anatomical structures and broader contextual relationship across CT slice. The Hybrid Identity Attention Block (HIA) module merges local detail with global context, ensuring that fine gland boundaries are preserved while maintaining consistency across all CT images. Finally, the Hybrid Bottleneck Attention Block (HBA) operates in the network’s central layers to efficiently connect global and local information, capturing important relationships in the image while keeping the model efficient and fast. Together, these modules improve key gland characteristics, preserve spatial information, and provide qualitative visual information that may assist interpretation of the model’s predictions while allowing the network to accurately detect parathyroid adenomas. This design seeks to improve the balance between computational efficiency, predictive performance, and qualitative model interpretability observed in prior works. Therefore, the proposed architecture provides a novel end-to-end framework that incorporates lightweight hierarchical attention mechanisms to facilitate qualitative interpretation of model predictions while achieving accurate parathyroid adenoma detection while bridging the gap between computational efficiency, diagnostic precision, and qualitative explainable AI. Beyond parathyroid adenoma localization, the framework contributes to the broader field of oncologic imaging by demonstrating how lightweight hierarchical attention mechanisms can support accurate lesion detection, provide qualitative insight into model predictions, and facilitate scalable deployment in precision diagnostic imaging. To achieve this, the present study has several key contributions as follows:

1. To curate an expert-annotated, high-quality dataset of arterial-phase CT images extracted from 4D-CT examination for benchmarking parathyroid adenoma (PTA) detection algorithms, ensuring consistent labeling and standardized acquisition parameters.
2. To develop four hierarchical attention components—namely, the Block-Level Attention Module (BLAM), Stage-Level Attention Module (SLAM), Hybrid Identity Attention block (HIA), and Hybrid Bottleneck Attention block (HBA)—that collectively enable multi-scale feature refinement, spatial context modeling, and efficient global–local information fusion.
3. To design, implement, validate and compare a family of 3 novel residual deep learning architectures optimized for arterial-phase CT imaging, integrating lightweight attention mechanisms for enhanced diagnostic precision.
4. To rigorously compare the proposed models against state-of-the-art ResNet50 architecture across multiple evaluation metrics, including accuracy, F1-score, and inference efficiency.
5. To evaluate the proposed residual attention-based framework in terms of diagnostic performance and computational efficiency, demonstrating its potential as a scalable framework for AI-assisted diagnostic detection.

This article’s remaining content is arranged as follows: a review of the relevant literature is given in Section 2. The methodology, including data collection, preprocessing, and the proposed architectures, is described in depth in Section 3. The experimental setup and the outcomes of the training, validation, and inference are presented in Section 4. The statistical analysis, computational cost, and a comparison with previous studies are covered in Section 5. Finally, Section 6 concludes the paper and outlines directions for future research.

## 2. Literature Review

Detection of parathyroid adenomas is crucial for successful minimally invasive parathyroidectomy. Although AI has demonstrated potential in automating CT analysis, existing approaches exhibit significant limitations. This review critically examines these gaps and limitations and establishes the foundation for our proposed methodology.

### 2.1. Foundations and Gaps in AI for Parathyroid Adenoma Detection

Although the application of AI to PTA detection has yielded encouraging results, the field is characterized by modality-specific developments that indicate a clear path for the work presented in this study. The majority of studies have focused on the analysis of nuclear medicine images, particularly 99mTc-sestamibi scintigraphy. Convolutional Neural Networks (CNNs) were shown to be useful in early research, with models like as ParaNet achieving high accuracy in distinguishing between normal and abnormal scans [12]. This progressed to more complex architectures, such as a three-path VGG19 network for multiphasic scintigraphy, which achieved a gland-specific sensitivity of 84.5% against surgical findings [13]. Since then, researchers have investigated explainability and transformer-based models. For instance, a Vision Transformer (ViT) applied to MIBI-early, MIBI-late, and Tc04 images produced a gland-level sensitivity of 93.85% [11], with subsequent work integrating Grad-CAM++ to visualize model attention [10]. Similarly, Yoshida et al. achieved lesion-based sensitivities of 82–83% on early- and late-phase scintigrams, though they highlighted the persistent challenge of managing false-positive rates [40]. Even though these studies demonstrate AI as a powerful tool for PTA detection, they exhibit recurring limitations that constrain their clinical applicability. Models are often tailored to a single, low-resolution modality (e.g., scintigraphy), which lacks the fine and subtle spatial information needed for precise anatomical localization. Furthermore, many state-of-the-art approaches, such as ViTs, are computationally demanding and require large datasets for effective generalization, making them ill-suited for the smaller cohorts typical of medical imaging studies. Additionally, the frequent trade-off between high sensitivity and acceptable false-positive rates remains a significant clinical challenge.

Researchers applying these techniques to intraoperative and advanced diagnostic modalities demonstrate that the application of AI extends beyond nuclear medicine. The analysis of near-infrared autofluorescence images has shown that healthy glands exhibit higher normalized intensity and reduced heterogeneity than diseased glands. Akbulut et al. used these features to train a decision-tree model that distinguishes normal from abnormal glands with 95% accuracy [41]. Avci et al. used autofluorescence images to train a visual deep learning network, achieving an AUROC of 0.90 and demonstrating the potential for real-time surgical guidance [42]. In parallel, Jarabek et al. applied a ResNet10-based model to FCH-PET images, accurately detecting hyperfunctioning parathyroid tissue in 83% of cases [43]. These studies demonstrate the adaptability of AI across diverse imaging modalities. However, progress remains highly modality-specific. Intraoperative systems like autofluorescence are designed for surface-level guidance and cannot support preoperative planning, while PET-based studies emphasize functional uptake over anatomical delineation. Models lack cross-modality generalization and are often trained on limited sample sizes. Notably, these approaches rely on ordinary CNNs or handcrafted features without taking advantage of integrated attention mechanisms to dynamically highlight discriminative regions during feature extraction.

To enhance the diagnostic reliability, several studies have gone beyond single-modality analysis by fusing imaging data with clinical and biochemical information. Yeh et al. combined 4D-CT and SPECT/CT findings with serum calcium and parathyroid hormone levels in a Random Forest model, achieving 90% accuracy on 458 patients [44]. Sandqvist et al. achieved an accuracy rate of 90% in identifying overlooked adenomas in multiglandular disease by a machine learning classifier with six predictor variables, including hormone levels and SPECT/CT results [17]. Similarly, Samaras et al. developed an explainable LightGBM model, which achieved 86.9% accuracy for adenomas using six clinical attributes to classify primary hyperparathyroidism subtypes [45]. While feature-level fusion improves the performance in these multimodal studies, they still rely on manual feature extraction from imaging and clinical reports. The rich spatial and temporal patterns inherent to modern imaging techniques are difficult to capture for traditional models like Random Forests and Gradient-Boosted Trees, and cannot perform end-to-end learning from raw, high-dimensional imaging data.

The most direct precedent for CT-based AI is the work by Greene et al. (2024), who used 4D-CT scans for the detection of PTA by comparing a Support Vector Machine (SVM) and a Bidirectional Long Short-Term Memory (Bi-LSTM) network [19]. Their Bi-LSTM model used the integrated morphological and temporal enhancement features across CT images and achieved an accuracy of 92%. This study provides a critical performance baseline, but instead of using modern, end-to-end convolutional architectures enhanced with attention, it relies only on sequential processing and conventional feature extraction. It leaves unexplored the potential of attention-based CNNs, which are not only effective but also powerful for image-based tasks.

### 2.2. The Evolution of Attention Mechanisms and Their Unexplored Potential in PTA Imaging

To address the challenges of complex medical image analysis, the field has increasingly turned to attention mechanisms, which enable networks to focus on subtle features. The following analysis examines their evolution and highlights their untapped potential for the specific task of PTA detection in 4D-CT.

The concept gained acceptance in natural language processing with the Transformer architecture [46] and was later modified and subsequently adapted for computer vision. A seminal adaptation, the Squeeze-and-Excitation Network (SENet), reduced the top-5 error on ImageNet to 6.62% by recalibration of channel-wise feature responses through modeling inter-channel dependencies [32]. Nevertheless, its global pooling operation eliminates local structural information by collapsing spatial dimensions. This is a crucial drawback for localizing small PTAs, where precise spatial information is essential for accurate diagnosis. Woo et al. proposed the Convolutional Block Attention Module (CBAM) to address this critical limitation by sequentially applying channel and spatial attention [33]. While CBAM improved object detection performance on benchmarks like MS-COCO, its sequential and independent computation of attention maps hinders the modeling of complex channel-spatial interdependencies. Furthermore, its reliance on pooling operations can still suppress fine details of small lesions.

Wang et al. introduced the Non-local Neural Network to capture global context by computing pairwise attention across all spatial positions [34]. Although this approach improved segmentation accuracy in video understanding, its computational complexity scales quadratically with the number of spatial positions. This computational burden is prohibitive for high-resolution 4D-CT volumes, often requiring aggressive downsampling that compromises the spatial precision needed to detect sub-centimeter parathyroid adenomas. This global self-attention paradigm was subsequently extended by the Vision Transformer (ViT), which treats image patches as input tokens and models their relationships using self-attention mechanisms [36]. While ViTs outperform the state-of-the-art on large-scale datasets like ImageNet, they lack the inherent inductive biases of CNNs for translation invariance and locality. This makes them prone to overfitting on smaller medical datasets and less effective for tasks requiring detailed localization due to their quadratic complexity and enormous data requirements [47].

Recent efforts have focused on improving efficiency. Huang et al. proposed the Criss-Cross Attention Network in an effort to reduce the complexity while maintaining segmentation accuracy by combining context along horizontal and vertical directions [35]. However, it is still largely a 2D operator and is not optimized for multi-dimensional volumetric data. To reduce computational complexity, Liu et al. proposed a probabilistic attention mechanism based on Laplace distributions [37]. Although efficient, these statistical approximations might limit robustness by oversimplifying the complex, heterogeneous appearance of PTAs across contrast phases. A comprehensive review by Xie et al. concluded that although the attention-based models improve the detection, they often suffer from high computational overhead on volumetric data, poor cross-modality generalization, and a lack of interpretability validation [39].

In summary, existing attention mechanisms present a fundamental trade-off: they either fail to efficiently capture long-range spatial and contextual dependencies or achieve this at the expense of preserving fine-grained spatial details. When applied to arterial-phase CT images for parathyroid adenoma detection, these limitations become more pronounced because accurate identification relies on subtle anatomical and contrast variations within small lesions. Despite these well-documented capabilities and trade-offs in the broader field, a survey of the parathyroid-specific literature confirms that these mechanisms have not been customized or applied to the challenge of PTA detection. Therefore, a distinct and unexplored opportunity exists to design lightweight, efficient attention mechanisms that can preserve local structural detail while guiding the model’s focus to diagnostically relevant regions across phases, specifically for the task of localizing parathyroid adenomas.

### 2.3. Synthesis and Identified Research Gap

The reviewed work, summarized in Table 1, demonstrates that while AI is a powerful tool for PTA detection, progress has been fragmented and has yet to incorporate a major advancement from computer vision: dedicated attention mechanisms. Studies on scintigraphy and other modalities achieve high accuracy but are constrained by their specific imaging physics and data characteristics, using models that lack focused feature enhancement. The most relevant prior work in 4D-CT [19] provides a baseline but relies on recurrent networks and traditional machine learning, not leveraging the spatial-feature prioritization of attention-based CNNs. Simultaneously, the field of computer vision offers a suite of powerful attention mechanisms, but these have not been translated to the specific challenges of PTA detection: small datasets, high-resolution volumetric data, and the critical need to localize small structures without sacrificing computational practicality.

This analysis reveals a convergent and previously unaddressed research gap: the complete absence of attention-enhanced deep learning models for parathyroid adenoma detection. This study is designed to fill this gap by being the first to introduce and tailor lightweight attention mechanisms for this task. We develop a family of residual deep learning networks integrated with custom, lightweight attention modules specifically optimized to leverage the rich spatial contextual data of arterial-phase CT. Our approach is novel in its aim to enhance focus on discriminative features for small adenomas while maintaining the computational efficiency required for integration into real-world clinical workflows upon validations.

## 3. Materials and Methods

**Figure 3:**
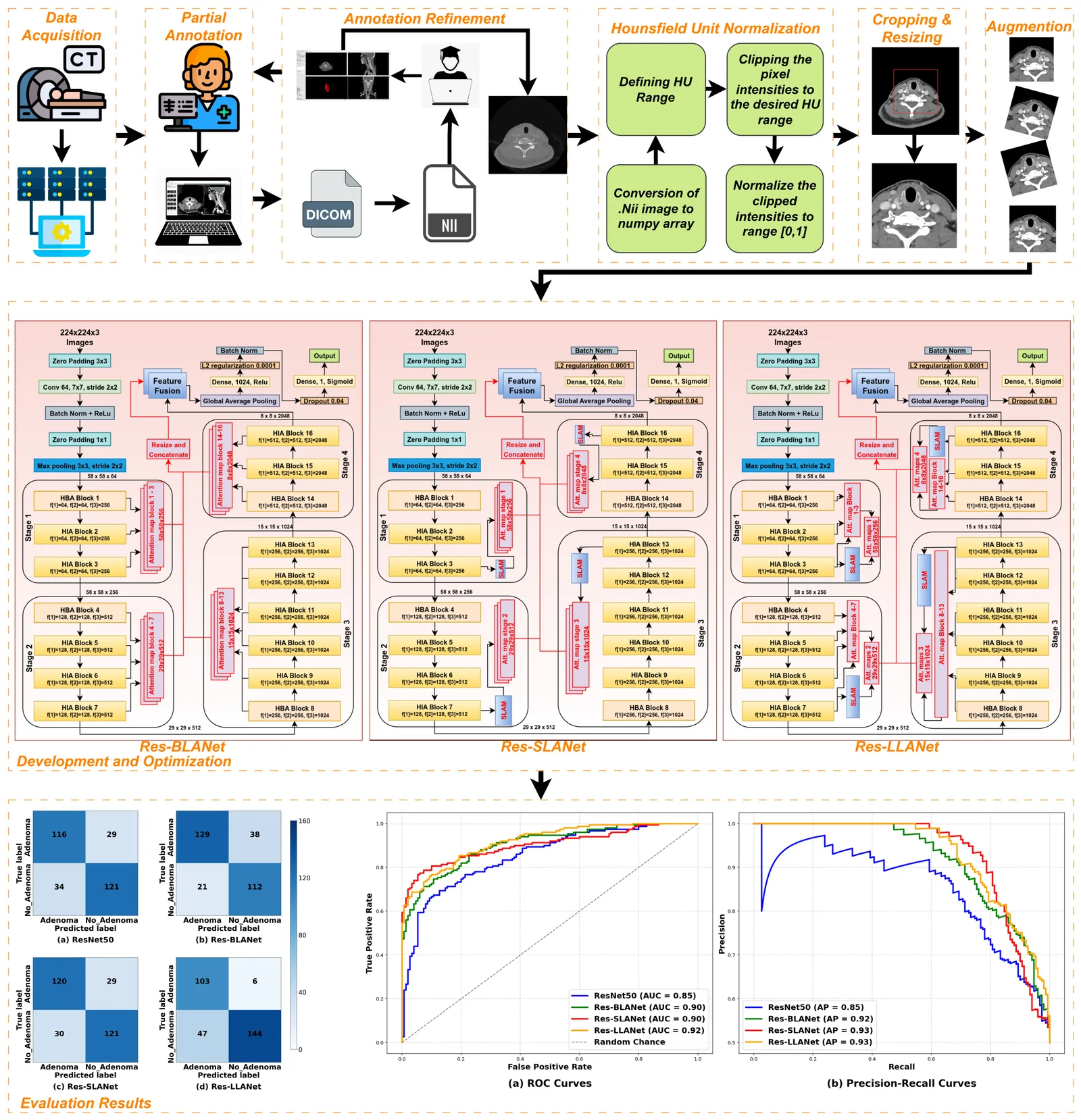
Methodological Summary.

This section details the comprehensive methodological framework developed for this study. Figure 3 provides an overview of the complete methodological workflow adopted in this study. The pipeline begins with data acquisition and partial clinical annotation of arterial-phase CT slices, followed by annotation refinement and conversion from DICOM to NIfTI format. Next, Hounsfield Unit (HU) normalization is performed by clipping voxel intensities to the defined HU range and scaling them into [0, 1]. The preprocessed slices are then cropped and resized to a uniform spatial dimension. In the second stage, the optimized architectures, Res-BLANet, Res-SLANet, and Res-LLANet, are developed and trained using these processed datasets. Finally, the evaluation phase includes quantitative comparisons across folds using confusion matrices, ROC and precision–recall curves, and training stability plots to assess classification robustness and generalization. We first describe the clinical dataset and the multi-step preprocessing pipeline designed to prepare the arterial-phase CT data for analysis. Subsequently, we provide a detailed explanation of the proposed deep learning architectures and their components.

### 3.1. Dataset and Preprocessing

**Figure 4:**
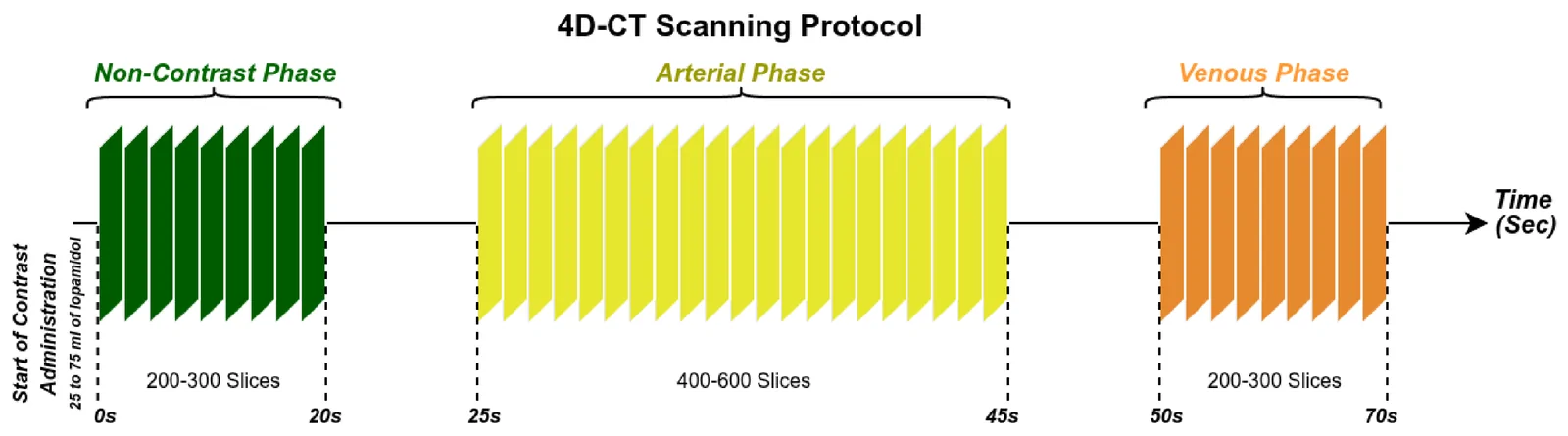
Schematic overview of the different phases in 4D-CT protocol.

The study group consisted of 63 patient cases evaluated by senior radiologists. This group included 39 patients with confirmed parathyroid adenoma. The diagnosis of each positive case was verified by pathological reports after surgical resection and was correlated with the CT findings. The remaining 24 cases showed no evidence of adenomas and served as the negative control group. The arterial-phase CT images for each patient were acquired using an Aquilion One Prism Edition scanner (Canon Medical Imaging Systems). The scanning protocol is illustrated schematically in Figure 4. It captured three distinct phases, beginning with a pre-contrast scan, followed by arterial and venous phases at approximately 25 and 50 s post-injection, respectively. The technical acquisition parameters were adjusted for each patient and included a tube voltage range of 80–135 kVp, an image matrix size of 512×512, a voxel resolution of 0.5×0.5×0.5 mm, and a pitch of 0.81 to 1.49. To improve image quality, 25 to 75 mL Iopamidol was administered intravenously as a contrast agent, with the specific volume determined based on the patient’s health status. Moreover, the data were collected retrospectively from clinical imaging archives, and all patient identifiers were permanently removed prior to analysis. Therefore, individual informed consent was not required under institutional guidelines for retrospective anonymized data use. The study protocol was reviewed and approved by the Kocaeli University Non-Interventional Clinical Research Ethics Committee (Approval No. E-26411792-020-597066). The research was conducted in accordance with the Declaration of Helsinki and national data protection regulations, ensuring that all ethical and privacy standards were strictly upheld.

Although the source imaging protocol was a multi-phase 4D-CT examination, only the arterial-phase CT images were extracted. The extracted arterial-phase CT images were stored in the standard DICOM format. To establish the ground truth for model training, a multi-step annotation process was performed by experienced radiologists. Using Sectra 7, radiologists first identified the key axial slices for each adenoma, including its initial appearance, its maximal size point, and the final slice before its disappearance. Following this initial localization, the open-source software ITK-SNAP version 4.2 was used to annotate all slices between the emergence and disappearance of the adenoma. To ensure the quality and consistency of the ground truth data, all labeled slices were subsequently re-evaluated by a senior radiologist before any further processing. The final annotations were then exported as binary image masks and stored in the NIfTI format, which is well-suited for deep learning applications.

Following the annotation process, the distribution of the annotated adenomas was reviewed in consultation with an experienced radiologist. The radiologist observed that parathyroid adenomas were consistently located within the lower cervical region of the arterial-phase CT examinations. Based on this clinical observation, a standardized slice-selection strategy was adopted. For each patient with a confirmed adenoma, 200 consecutive slices covering the annotated lesion region were extracted. For negative patients, 200 consecutive slices were selected from the anatomically corresponding lower cervical region, following the same clinically relevant anatomical location recommended by the radiologist. This strategy was adopted to ensure that both positive and negative samples were drawn from comparable anatomical regions while excluding anatomically irrelevant portions of the CT volume. Although this clinically guided sampling reduces large anatomical differences between the two classes, we acknowledge that it cannot completely eliminate the possibility of positional or sampling bias.

**Figure 5:**
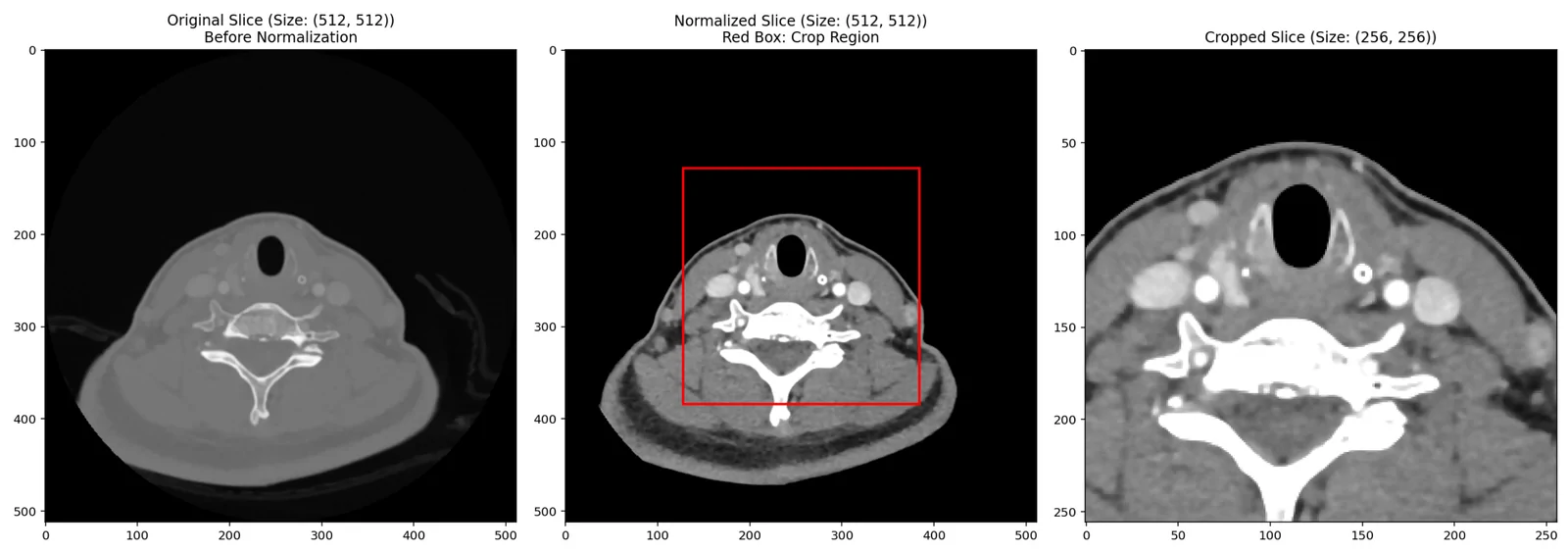
Normalization and cropping step performed in the preprocessing on the dataset. Showed example Patient 1 with slice index 260. The red box indicates the region of interest (ROI) that was cropped for subsequent analysis.

Once the dataset was curated, the selected image slices were passed through a multi-step preprocessing pipeline. Direct analysis of the raw images was challenging because their inherent contrast often diminished when processed, making it difficult to discern subtle tissue differences. Consequently, the Hounsfield Unit (HU) normalization approach was applied as the primary contrast enhancement technique. The process began by applying a specific HU window of [−200, 300]. This step served to isolate the relevant soft tissue structures of the neck, where adenomas are located, while simultaneously excluding diagnostically irrelevant information such as the low density of air or the high density of bone. The pixel values within this clinically relevant window were then rescaled to a floating point range using the min-max normalization formula shown in Equation (1) [48]. This process is critical as it preserves the essential tissue contrasts while ensuring a uniform intensity representation across images from different patients and acquisitions. Finally, a central crop was applied to each slice, reducing its dimensions to the 224×224 pixels required by the network architecture. This step served a dual purpose. Firstly, it enhanced computational efficiency by reducing the input data size. Secondly, and more critically, it served to amplify the target anatomical region. Since parathyroid adenomas are located centrally within the neck, this crop effectively removes peripheral, non-informative areas, forcing the model to concentrate its learning on the central structures where adenomas are likely to be found [49]. The result of the applied preprocessing pipeline is visualized in Figure 5.

(1) $$I_{\mathrm{normalized}}=\frac{I-{\mathrm{HU}}_{\min}}{{\mathrm{HU}}_{\max}-{\mathrm{HU}}_{\min}}$$

For each patient, 200 arterial-phase CT slices were extracted according to the preprocessing protocol. To improve data diversity and model generalization, an offline data augmentation strategy was applied exclusively to the training dataset. For each patient, an additional set of 200 augmented images was generated by randomly applying horizontal flipping (probability = 0.5), scaling (0.9), rotation (±15°), random brightness and contrast adjustment (±20%) and Gaussian blur (kernel size 3×3, σ=0.3). The augmentation parameters were randomly sampled for each generated image, producing diverse transformed versions while preserving the underlying anatomical structures. Consequently, each training patient contributed 200 original and 200 augmented images (400 images in total). Data augmentation was not applied to the validation or independent test datasets.

### 3.2. Proposed Architecture

The proposed architectures enhance a ResNet50 backbone by integrating two custom-designed, computationally efficient attention modules, a Block-level Light Attention Module (BLAM) and a Stage-level Light Attention Module (SLAM). These lightweight modules were developed to improve feature representation and capture crucial long-range dependencies in the feature maps, without incurring the high computational cost of standard self-attention mechanisms. The following sections first detail these core components and then describe how they are systematically integrated to construct our three proposed networks, Res-BLANet, Res-SLANet, and Res-LLANet.

Unlike conventional attention modules that can be incorporated into an existing CNN backbone as independent plug-in components, the proposed BLAM and SLAM are structurally integrated within the residual learning framework. Specifically, BLAM is embedded inside the proposed Hybrid Bottleneck Attention (HBA) and Hybrid Identity Attention (HIA) residual blocks, whereas SLAM operates on the output feature maps of entire residual stages. Furthermore, the hierarchical feature fusion employed in Res-LLANet is an inherent architectural component that combines the original residual features with the corresponding attention-enhanced representations before subsequent processing. Consequently, the attention modules and the feature fusion mechanism are mutually dependent design components rather than independent modules that can be removed or evaluated separately without fundamentally altering the proposed network architecture.

#### 3.2.1. Attention Modules (BLAM & SLAM)

**Figure 6:**
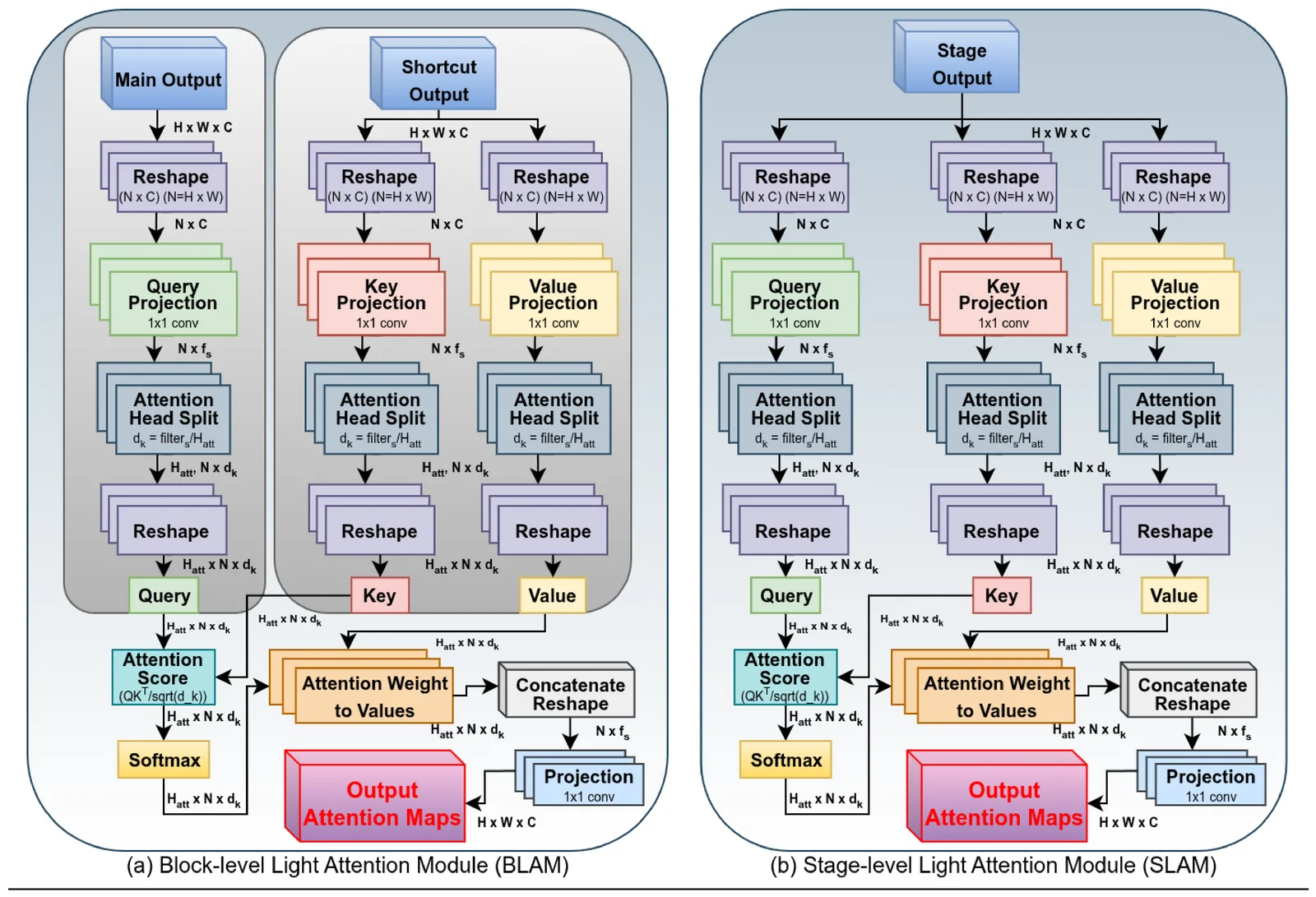
Architecture of BLAM and SLAM.

To improve feature representation within our residual learning framework, we developed two efficient attention mechanisms, the Block-level Light Attention Module (BLAM) and the Stage-level Light Attention Module (SLAM). The architectures for both modules are illustrated in Figure 6. These mechanisms are designed to incorporate the powerful principles of multi-scale attention while preserving computational efficiency. Their primary objective is to enhance the model’s ability to capture both fine-grained local features and broader global contextual information. By achieving this without a significant increase in computational cost, they function as light attention mechanisms, making them highly suitable for complex medical imaging tasks. A significant barrier to using conventional self-attention in computer vision is its computational demand. The standard mechanism calculates the pairwise similarity between all spatial coordinates (N=H×W), resulting in a computational complexity of $O(N^{2})$. This quadratic scaling is prohibitively expensive for the high-resolution feature maps commonly found in medical imaging. The proposed light attention modules mitigate this problem through several key optimizations. First, to reduce the cost of the dot-product operation, we project the queries, keys, and values into a lower-dimensional feature space ($f_{s}$) using efficient 1×1 convolutions before the attention calculation begins. Second, to further minimize computational load, these reduced features are divided among multiple attention heads, each operating on a smaller per-head dimensionality ($d_{k}$). Third, to avoid redundant processing, attention is applied selectively at either the residual block level (BLAM) or the stage output level (SLAM), rather than at every layer of the network. Lastly, the modules employ reshaping operations between the spatial feature map (H×W×C) and flattened sequence (N×C) formats to prevent unnecessary data transformations. These strategies collectively reduce the computational burden, making the benefits of self-attention practical for our task.

The BLAM module operates within a residual block and is designed to process two parallel inputs: the Main Output from the block’s convolutional path and the Shortcut Output from its skip connection. Both inputs are tensors with the shape H×W×C. The process begins with the generation of the Query matrix (*Q*). The Main Output tensor is first transformed from a spatial feature map (H×W×C) into a flattened sequence (N×C), where N=H×W. This transformation converts the 2D spatial grid into a series of *N* tokens, each having *C* (or $f_{s}$ after projection) features. This sequence is then passed through a 1×1 convolutional layer, which functions as a projection to produce the final query matrix, $Q\in R^{N\times f_{s}}$. The term $f_{s}$ represents the reduced feature dimension after this projection. To produce the Key (*K*) and Value (*V*) matrices, the Shortcut Output is split into two parallel streams. Each stream undergoes a similar reshaping and projection process using distinct 1×1 convolutional layers, resulting in $K,V\in R^{N\times f_{s}}$. The feature dimension $f_{s}$ is then divided among multiple attention heads ($H_{\mathrm{att}}$) to yield a per-head dimensionality, $d_{k}$, as defined in Equation (2). This creates Query, Key, and Value tensors with dimensions $H_{\mathrm{att}}\times N\times d_{k}$. Each head independently calculates an attention score using the scaled dot-product attention mechanism, which is then normalized with a softmax function to produce attention weights, as shown in Equation (3). The outputs from all attention heads are then concatenated, reshaped back to $N\times f_{s}$, and passed through a final 1×1 convolutional layer. This produces the final attended feature map with the original shape of H×W×C. By processing the shortcut link in this way, BLAM enables the network to selectively focus on information that is complementary to the features passed along the main pathway and the residual pathway.

(2) $$d_{k}=\frac{f_{s}}{H_{\mathrm{att}}}$$

(3) $$\mathrm{Attention}\left(Q,K,V\right)=\mathrm{Softmax}\left(\frac{QK^{T}}{\sqrt{d_{k}}}\right)V$$

In contrast to the block-level operation of BLAM, the SLAM module functions at the end of an entire architectural stage and employs a comparable computational flow. The primary distinction is its input source. Instead of using separate main and shortcut pathways, SLAM takes the final Stage Output feature map as its single input to generate the queries, keys, and values. To create the *Q*, *K*, and *V* matrices, the input tensor (H×W×C) is first reshaped into a flattened sequence (N×C). This sequence is then projected using three distinct 1×1 convolutional layers to produce $Q,K,V\in R^{N\times f_{s}}$. From this point, the process mirrors that of BLAM. The features are split into multiple attention heads, the scaled dot-product attention equation is applied, and the multi-scale outputs are concatenated and projected to reconstruct the final attended feature map. This structural difference leads to a fundamental conceptual distinction between the two modules. BLAM is designed to capture localized, complementary features between a block’s output and its shortcut connection, thereby enhancing fine-grained interactions. In contrast, SLAM captures global attention features across an entire stage’s feature map. This allows SLAM to model more general, long-range contextual relationships within the data, complementing the local refinement provided by BLAM.

#### 3.2.2. Attention-Enhanced Blocks (HBA & HIA)

To directly integrate our attention mechanism into the feature extraction backbone of the network, we incorporated the Block-level Light Attention Module (BLAM) into the residual blocks. This integration resulted in the development of two novel, attention-enhanced modules that form the primary building blocks of our proposed architectures, the Hybrid Bottleneck Attention (HBA) Block and the Hybrid Identity Attention (HIA) Block.

**Figure 7:**
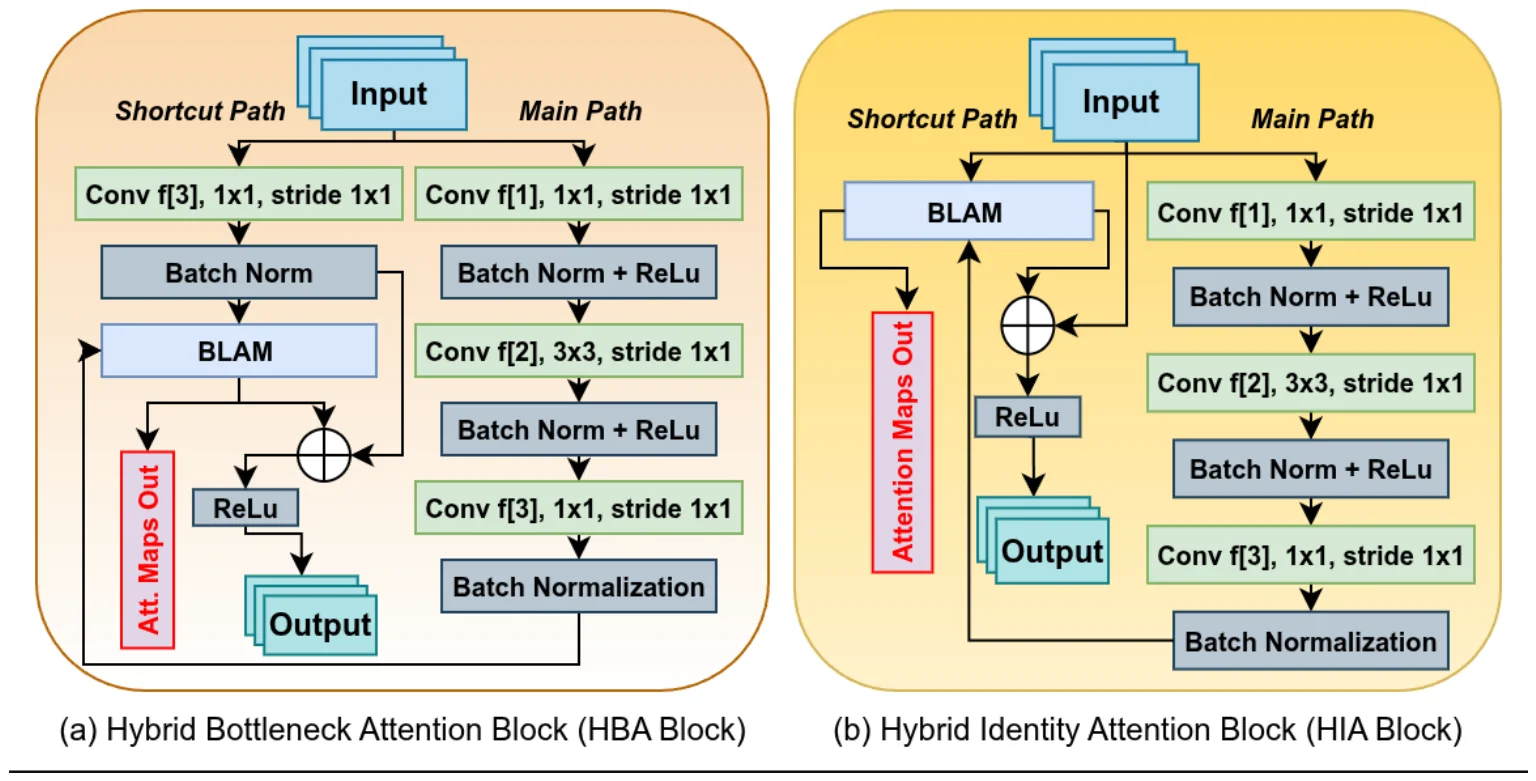
Architecture of HBA and HIA Blocks.

The HBA Block, shown in Figure 7a, processes an input feature map along two parallel paths. The main path follows a standard bottleneck convolutional structure. It consists of a 1×1 convolution for channel reduction, a 3×3 convolution for spatial feature extraction, and a final 1×1 convolution to restore the original channel dimensions. Each convolutional layer is followed by batch normalization and a ReLU activation. In parallel, the shortcut path is designed to generate an attention-refined version of the input. It first applies a 1×1 convolution to the input feature map, which ensures that its channel dimensions match the output of the main path. This projected feature map is then processed by the BLAM module, which generates attention maps that highlight the most informative regions. The final representation for the HBA block is produced by summing the outputs of the main and shortcut paths element-wise, followed by a final ReLU activation. This architecture effectively combines a powerful feature transformation with an attention-driven feature refinement.

The HIA Block, illustrated in Figure 7b, utilizes a similar two-path structure but with a critical architectural difference. Instead of a convolution-based shortcut, the HIA Block employs a true identity shortcut, where the input feature map is passed along the shortcut path without any initial transformation. In this configuration, the BLAM module is applied directly to these identity-mapped features. This allows the attention mechanism to work directly on the original, unmodified features from the block’s input. The main path of the HIA Block retains the same effective bottleneck convolutional sequence used in the HBA Block to ensure efficient feature extraction. The final output is produced by adding the output of the main path to the BLAM enhanced shortcut path, followed by a ReLU activation. By adaptively re-weighting the original feature identity with attention maps, the HIA Block improves contextual modeling with minimal additional computational expense.

By combining the BLAM with either a bottleneck convolutional path (HBA) or an identity shortcut path (HIA), our proposed modules strike an effective balance between discriminative feature highlighting and computational efficiency. This makes them well-suited as foundational building blocks for deep network architectures designed for high resolution visual recognition tasks. Crucially, the attention maps generated within each HBA and HIA block are retained. This output is later used in a feature fusion step, a strategy that helps the model to effectively identify the spatial areas for parathyroid adenoma diagnosis. This hybrid methodology combines the spatial feature extraction capabilities of CNNs with the dynamic feature weighting strengths of attention, creating a powerful and efficient framework for medical imaging analysis. These residual blocks are used to construct three distinct models, with each model varying based on how the attention modules are integrated into the broader network.

#### 3.2.3. Multi-Scale Attention Fusion

**Figure 8:**
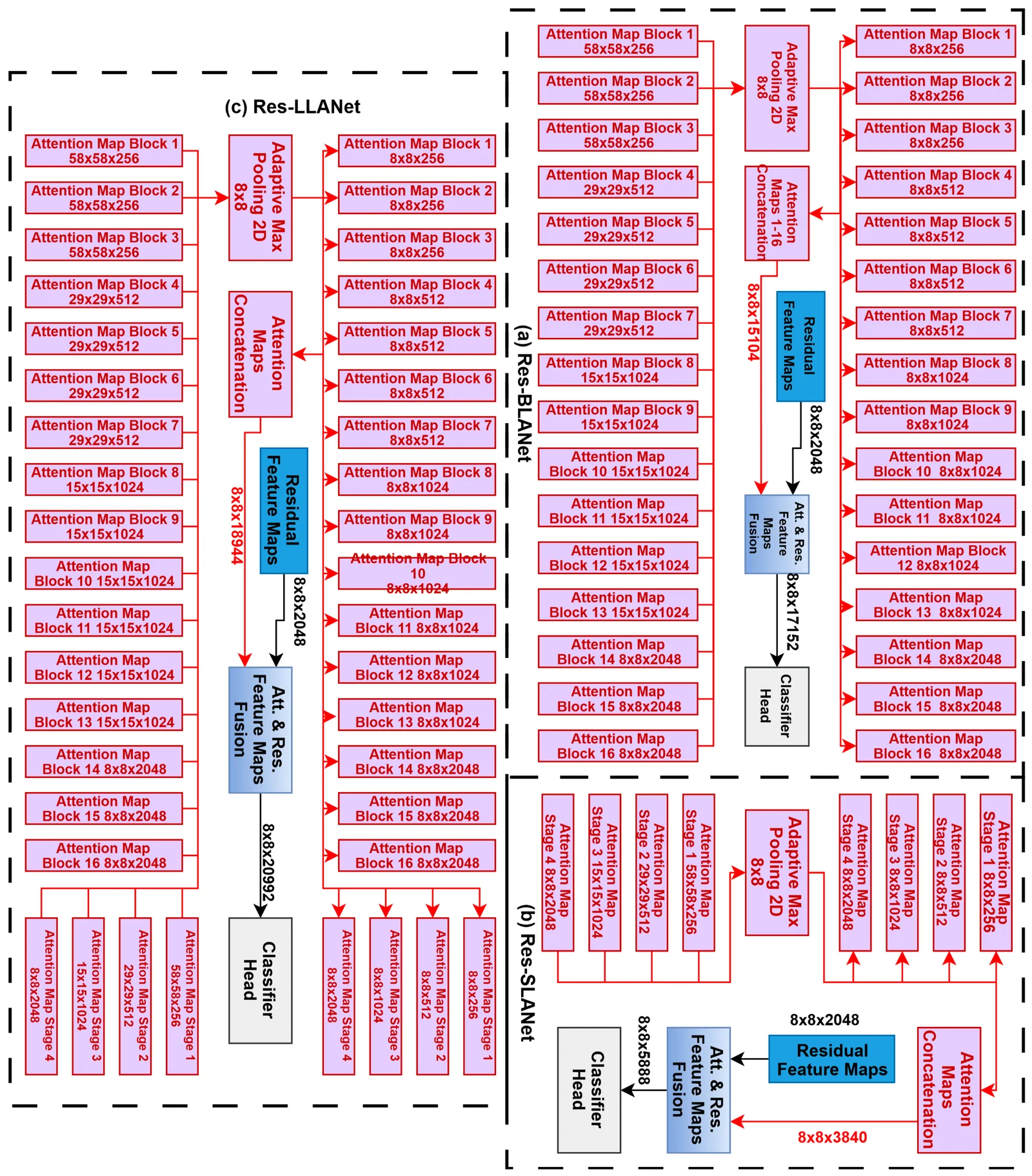
Architectural overview of the resize, concatenation, and fusion mechanism of the proposed networks: (**a**) Res-BLANet, (**b**) Res-SLANet, and (**c**) Res-LLANet.

A core component of our architectural strategy is the aggregation of information from the attention maps generated at various levels of the network. As input data propagates through the network, the attention maps capture contextual information at progressively different scales, from fine-grained details in early layers to high-level semantic features in deeper layers. To leverage this rich, multi-scale information, we developed a fusion mechanism, illustrated in Figure 8, which combines these distributed attention maps into a comprehensive feature representation for the final classifier. The fusion process begins by standardizing the spatial dimensions of the attention maps. Because the attention maps are generated by blocks or stages at different depths, they have varying spatial resolutions.

To achieve efficient multi-scale feature integration, each attention map generated at different stages of the network (e.g., 58×58, 29×29, 15×15, and 8×8) is first normalized to a uniform spatial resolution of 8×8 using an Adaptive Max Pooling operation. Unlike conventional pooling, which applies fixed kernel and stride values, adaptive pooling automatically determines these parameters so that the output size remains constant, regardless of the input dimensions. Mathematically, for an input feature map of spatial size $\left(H_{in},W_{in}\right)$ and a target output size $\left(H_{out},W_{out}\right)$, The Adaptive Max Pooling operation divides the input into approximately equal regions. Each output cell (i,j) is computed over the input ranges:

(4) $${\mathrm{start}}_{i}=\left\lfloor \frac{i\times H_{in}}{H_{out}}\right\rfloor ,\;{\mathrm{end}}_{i}=\left\lceil \frac{\left(i+1\right)\times H_{in}}{H_{out}}\right\rceil ,$$

(5) $${\mathrm{start}}_{j}=\left\lfloor \frac{j\times W_{in}}{W_{out}}\right\rfloor ,\;{\mathrm{end}}_{j}=\left\lceil \frac{\left(j+1\right)\times W_{in}}{W_{out}}\right\rceil .$$

The output value at position (i,j) is then obtained as:

(6) $$y_{i,j}=\max_{h\in \left[{\mathrm{start}}_{i},\;{\mathrm{end}}_{i}\right),\;w\in \left[{\mathrm{start}}_{j},\;{\mathrm{end}}_{j}\right)}x_{h,w}.$$

This formulation guarantees that the output dimensions remain fixed at $\left(H_{out},W_{out}\right)$ regardless of the input resolution, ensuring consistent multi-scale aggregation across varying attention map sizes.

This mechanism ensures that all feature maps, regardless of their original resolution, are adaptively downsampled to a consistent 8×8 spatial domain while preserving their most salient activations. Once unified in scale, the attention maps are concatenated along the channel dimension to form a comprehensive tensor:

(7) $$F_{\mathrm{concat}}=\left[A_{1}^{8\times 8},A_{2}^{8\times 8},\ldots ,A_{n}^{8\times 8}\right],$$

where $A_{i}^{8\times 8}$ denotes the downsampled attention map from the $i^{th}$ stage.

While adaptive pooling inevitably compresses spatial details, potential information loss is mitigated through a residual attention fusion mechanism. The original residual feature maps $R\in R^{8\times 8\times C_{r}}$ (where, $C_{r}$ is the no. of channels of residual feature maps) obtained from the backbone are concatenated with the aggregated multi-scale attention features $A\in R^{8\times 8\times C_{a}}$ (where, $C_{a}$ is the no. of channels of attention feature maps) along the channel axis:

(8) $$F_{\mathrm{fusion}}=\mathrm{Concat}\left(R,A\right),$$

resulting in a unified tensor $F_{\mathrm{fusion}}\in R^{8\times 8\times (C_{r}+C_{a})}$. This channel-wise fusion strategy preserves the discriminative power of both representations; the residual features contribute deep semantic consistency, while the attention features encode fine-grained spatial saliency across scales.

By maintaining all learned feature responses instead of averaging them, the network minimizes attention dilution and enhances sensitivity to subtle intensity and morphological variations. This operation stacks the feature maps from different blocks or stages into a single, high-dimensional tensor, effectively combining all the distributed attention information into one unified representation. The dimensions of this concatenated tensor vary depending on the specific model architecture. For example, the Res-BLANet, which aggregates attention maps from all 16 of its residual blocks, produces a tensor with a size of 8×8×15,104. In contrast, the Res-SLANet concatenates only the four stage-level attention maps, resulting in a more compact tensor of 8×8×3840. The Res-LLANet expands on this concept by combining all local-level attention maps, creating the largest aggregated tensor with a size of 8×8×18,944. The final step in the process is the Attention and Residual Feature Maps Fusion phase. In this stage, the large, concatenated attention tensor is fused with the final residual feature map produced by the network’s backbone (e.g., the 8×8×2048 feature map from the last stage). This fusion creates a highly discriminative representation that is then passed to the final classification head. The strategic benefit of this approach is significant. By mixing the fine-grained details captured by early-layer attention maps with the high-level semantic information from deeper layers, the model can make a more informed and robust classification. This ability to efficiently collect and integrate multi-scale contextual information is a key factor in boosting the model’s feature discriminability and overall performance.

#### 3.2.4. Residual Block Light Attention Network (Res-BLANet)

**Figure 9:**
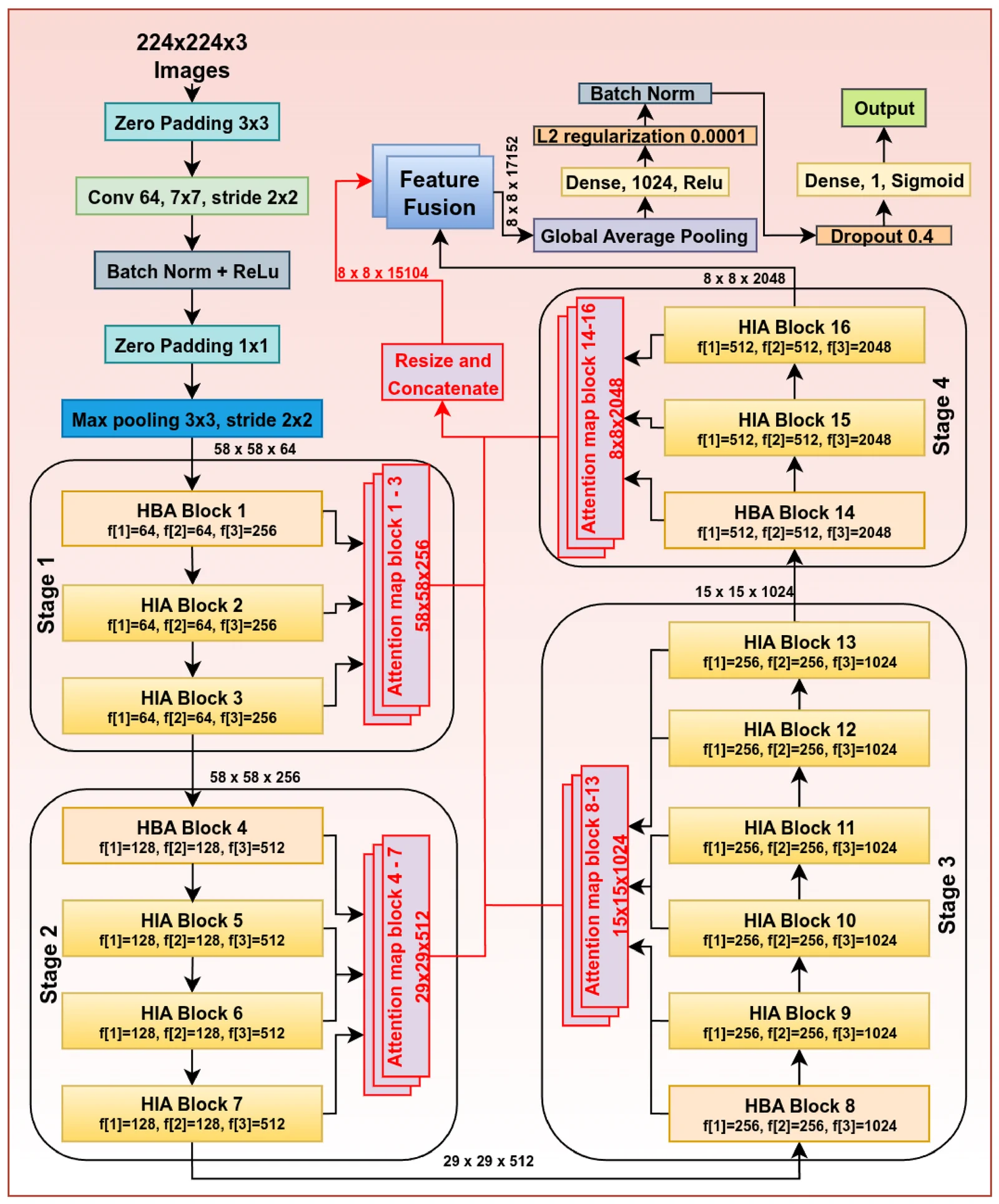
Res-BLANet Design.

The first proposed model, the Residual Block Light Attention Network (Res-BLANet), is designed to enhance feature representation by deeply integrating attention at the most granular level of the network. As illustrated in the detailed architectural diagram in Figure 9, the core principle of Res-BLANet is the incorporation of the Block-level Light Attention Module (BLAM) within every residual block of its deep convolutional framework.

The network’s initial feature extraction stem processes an input image of size 224×224×3. The input first passes through a 3×3 zero-padding layer, followed by a 7×7 convolution with 64 filters and a stride of 2. After batch normalization and a ReLU activation, the resulting feature map is passed through a 3×3 max-pooling operation, also with a stride of 2. This sequence of operations effectively downsamples the input and produces an initial feature map with dimensions of 58×58×64.

The main body of the network is divided into four successive stages, each composed of a specific combination of the Hybrid Bottleneck Attention (HBA) and Hybrid Identity Attention (HIA) blocks.

- Stage 1 begins the deep feature extraction with one HBA block followed by two HIA blocks. The channel configuration for this stage is f[1]=64, f[2]=64, and f[3]=256.
- Stage 2 increases the feature complexity with one HBA block and three HIA blocks, enlarging the channel dimensions to f[1]=128, f[2]=128, and f[3]=512.
- Stage 3 further deepens the network with one HBA block and five HIA blocks, increasing the channel configuration to f[1]=256, f[2]=256, and f[3]=1024.
- Stage 4 forms the final feature extraction phase with one HBA block and two HIA blocks, employing the highest channel configuration of f[1]=512, f[2]=512, and f[3]=2048.

A crucial feature of the Res-BLANet architecture is its use of the multi-scale attention fusion strategy previously described. By resizing and concatenating the attention maps produced by the BLAM module in each of the 16 residual blocks, the network creates a rich, multi-scale representation of the most salient image features. This fused representation is then processed by the classification head. The process begins with a global average pooling layer, which reduces the final feature map from Stage 4 to a 1×1×2048 vector. This is followed by a fully connected layer with 1024 units, a ReLU activation, and L2 regularization (λ=0.0001). A dropout layer with a rate of p=0.04 is applied for further regularization before the final dense layer. This output layer consists of a single neuron with a sigmoid activation function for binary classification. This design successfully blends the effectiveness of bottleneck residual blocks with the fine-grained discriminative power of block-level light attention, creating a model well suited for accuracy-critical vision tasks.

#### 3.2.5. Residual Stage Light Attention Network (Res-SLANet)

**Figure 10:**
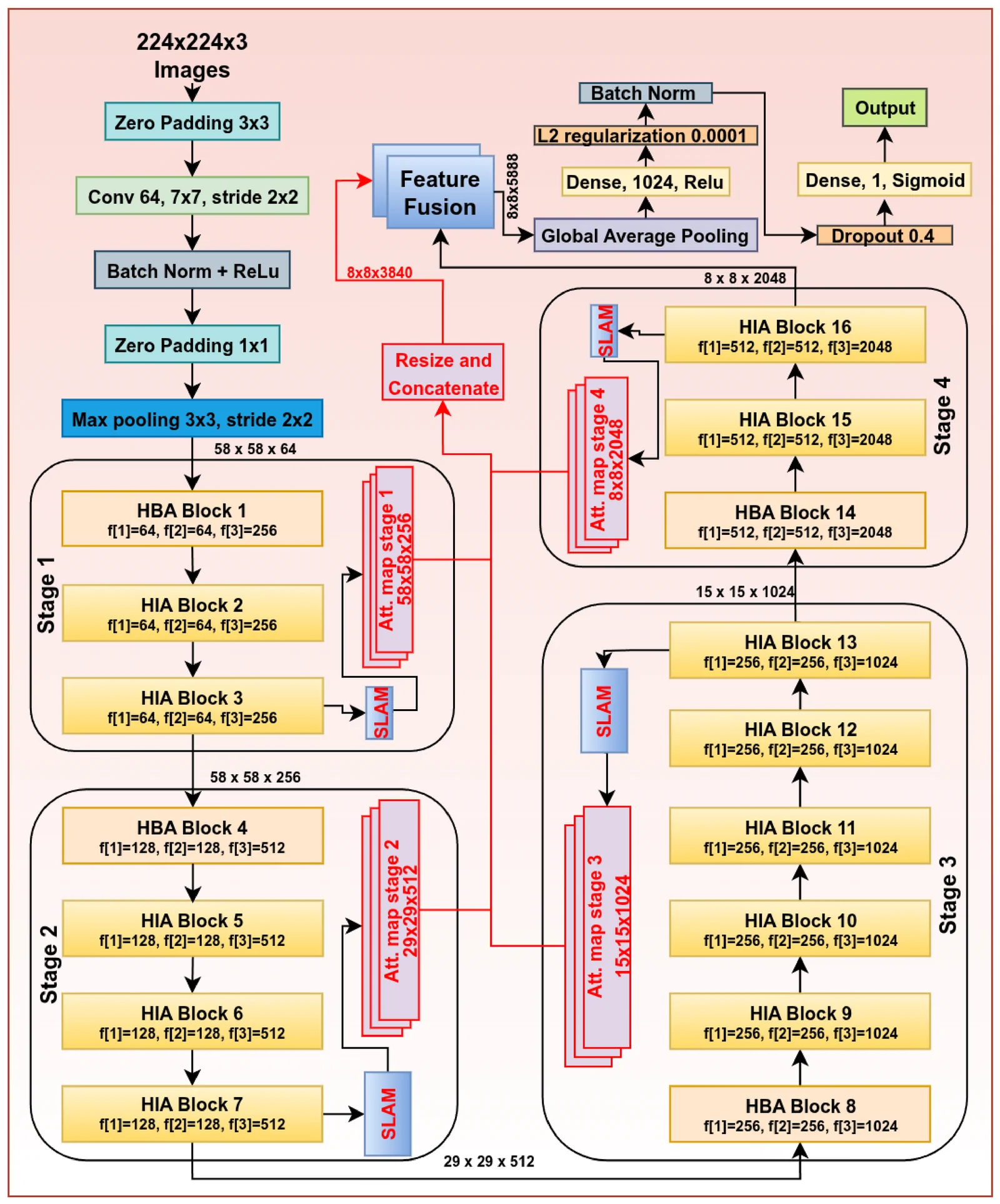
Res-SLANet Design.

The second proposed model, the Residual Stage Light Attention Network (Res-SLANet), is detailed in Figure 10. In contrast to Res-BLANet, which integrates attention at a granular block level, this architecture implements a lightweight multi-scale attention module at the end of each major ResNet stage. This strategic shift enables the network to acquire a more extensive global context across its increasingly abstracted feature representations. The fundamental ResNet50 architecture is maintained as the feature extraction backbone of the model. This includes the standard sequence of convolutional and identity blocks, complete with the residual connections that are essential for efficient gradient flow in deep networks. The key innovation in Res-SLANet lies in how attention is applied. Following each of the four stages in the ResNet backbone, the final output feature map is passed to a bespoke lightweight Multi-Scale Attention, which implements the Stage-level Light Attention Module (SLAM). Within this module, the output feature map of each stage is first reshaped from its spatial dimensions into a 2D sequence. This sequence is then processed by the scaled dot-product multi-scale attention mechanism, which allows the model to identify non-local dependencies and highlight semantically significant regions across the entire feature map. Following the attention calculation, the attended outputs are reformed back to their original spatial dimensions. To create a single, context-aware representation, the attended feature maps from all four stages are aggregated. Due to the progressive downsampling in the ResNet backbone, the attention outputs from the earlier stages have larger spatial resolutions. Therefore, these outputs are first downscaled using adaptive max pooling to match the dimensions of the final stage’s feature map. These four resized, attention-refined maps are then concatenated along the channel axis with the final feature map from the network’s backbone. This stage-level attention and fusion method allows the model to effectively consolidate multi-scale contextual information while preserving computational efficiency. It enhances the native spatial feature extraction of the convolutional layers by globally refining the most essential characteristics identified across the network’s various levels of abstraction.

#### 3.2.6. Residual Layer Light Attention Network (Res-LLANet)

**Figure 11:**
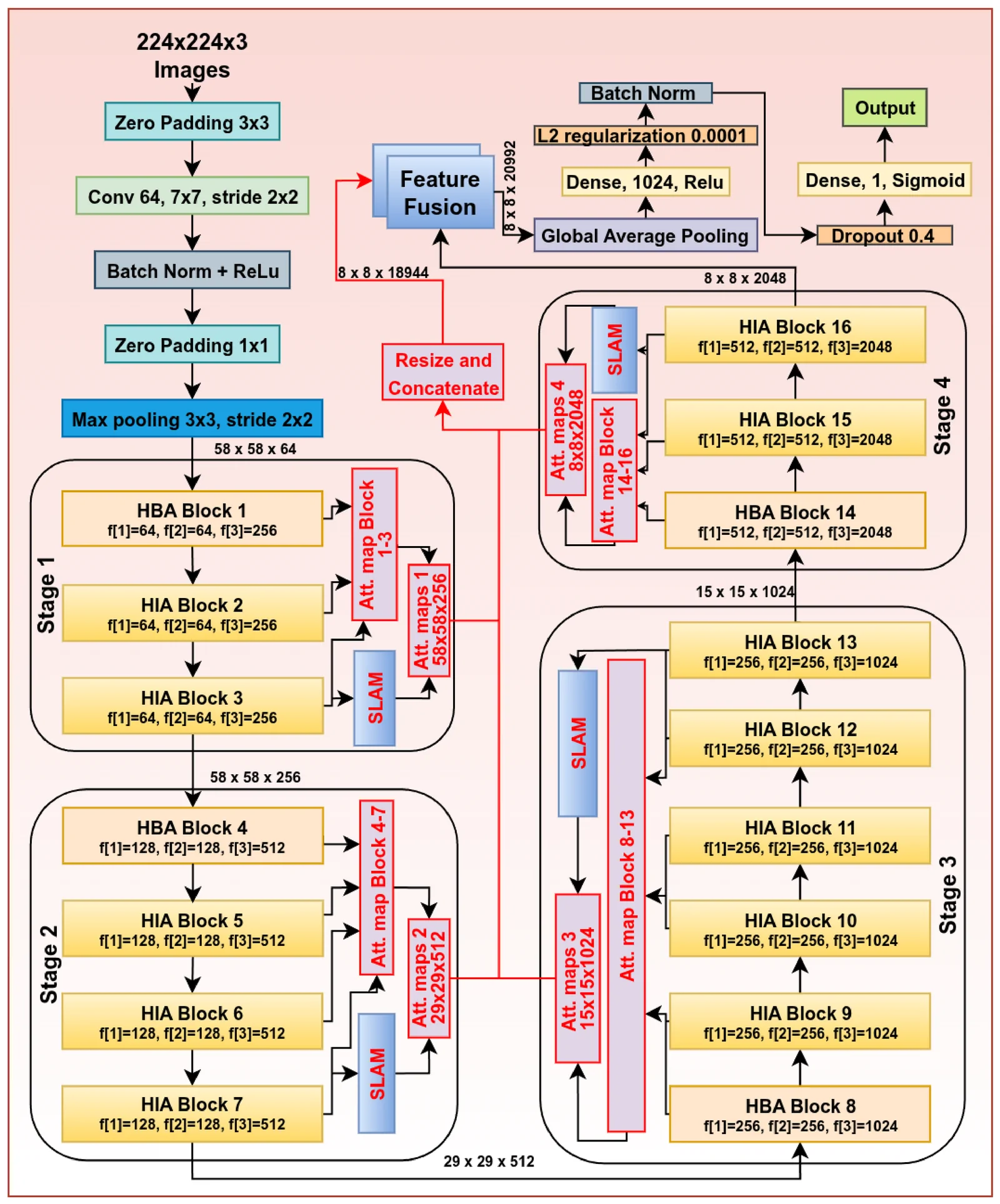
Res-LLANet Design.

The third proposed model, the Residual Layer Light Attention Network (Res-LLANet), features a hybrid attention augmented architecture, as shown in Figure 11. The core innovation of this model is its dual integration technique, which is designed to merge the advantages of both fine-grained local attention and global contextual attention. By combining the strategies of the previous two models, Res-LLANet aims to maximize feature expressiveness and sensitivity for the challenging task of parathyroid adenoma identification. First, the model preserves the ResNet50 architecture with its standard convolutional and identity blocks. Similar to Res-BLANet, this model enhances these blocks by incorporating multi-head attention layers between the final convolutional layer and the residual addition step. This block-level attention enables the network to dynamically enhance both channel-wise and spatial feature representations directly within the residual flow. This process facilitates the model’s ability to focus on more discriminative local patterns as features are being extracted. In addition to this fine-grained, block-level integration, the model also incorporates stage-level multi-scale attention aggregation, similar to the strategy employed by Res-SLANet. The output from the final block of each of the principal ResNet stages (e.g., conv2 block3, conv3 block4, etc.) is passed through a dedicated multi-scale attention mechanism. This allows the model to capture global, stage-wide contextual information at multiple levels of feature abstraction. The outputs from these stage-level attention mechanisms are gathered for final fusion. To address the varying spatial resolutions that result from downsampling, each attention map is first downsampled to correspond with the final stage’s resolution using an adaptive max pooling layer. These resized attention maps are then concatenated with the primary network’s final output before being transmitted to the classification head. By integrating attention at both the block and stage levels, this model is designed to grasp intricate, multi-scale global dependencies while simultaneously refining discriminative local patterns. This dual-integration approach aims to create the most expressive and sensitive feature representations, combining the strengths of both local and global attention mechanisms across several levels of abstraction.

## 4. Results

### 4.1. Evaluation Metrics

Evaluation metrics are crucial for evaluating the effectiveness of machine learning models. The confusion matrix, which classifies the model’s predictions into True Positives (TP), False Positives (FP), True Negatives (TN), and False Negatives (FN), is one of the key measures. Derived measures like accuracy, precision, recall, and F1-score provide additional insights into how effective the model is [50]. Furthermore, the capacity of a model to distinguish between classes is measured by the ROC curve (Receiver Operating Characteristic) and the corresponding AUC (Area Under the Curve) [51].

(9) $$\mathrm{Accuracy}=\frac{\mathrm{TP}+\mathrm{TN}}{\mathrm{TP}+\mathrm{TN}+\mathrm{FP}+\mathrm{FN}}$$

(10) $$\mathrm{Precision}\;\left(\mathrm{PPV}\right)=\frac{\mathrm{TP}}{\mathrm{TP}+\mathrm{FP}}$$

(11) $$\mathrm{Recall}\;\left(\mathrm{TPR}\right)=\frac{\mathrm{TP}}{\mathrm{TP}+\mathrm{FN}}$$

(12) $$F1\_\mathrm{score}=2\cdot \frac{\mathrm{Precision}\cdot \mathrm{Recall}}{\mathrm{Precision}+\mathrm{Recall}}$$

### 4.2. Experimental Setup

The dataset was divided at the patient level using a 10-fold cross-validation strategy to prevent data leakage between folds while preserving the class distribution. Prior to cross-validation, three patients (two adenoma-positive and one adenoma-negative) were held out as an test set and were excluded from all stages of model development. The remaining 60 patients (37 adenoma-positive and 23 adenoma-negative) were used for 10-fold cross-validation. In each fold, 54 patients (34 adenoma-positive and 20 adenoma-negative) were used for training, whereas the validation set consisted of six patients (three adenoma-positive and three adenoma-negative). The patient distribution across the dataset partitions is summarized in Table 2. The test set was used only for the final performance evaluation and was not involved in training, validation, hyperparameter optimization, or model selection. Owing to the limited number of pathologically confirmed parathyroid adenoma cases satisfying the inclusion criteria, only three patients were reserved for independent testing, while the remaining patients were utilized within the cross-validation framework to maximize the available training data.

Prior to the final experiments, a manual grid search was conducted exclusively on the training data within the 10-fold cross-validation framework to optimize the training hyperparameters. Hyperparameter optimization was performed for the initial learning rate, dropout rate, and L2 weight regularization coefficient. The optimal hyperparameter combination was selected based on the highest mean validation F1-score across the cross-validation folds. The independent test cohort was not used during hyperparameter optimization. The evaluated search space and the selected hyperparameters are summarized in Table 3.

Model training was performed using the optimal hyperparameter configuration obtained from the manual grid search (Table 3). Binary cross-entropy with class-balancing weights was employed as the loss function. The attention modules were configured with eight attention heads, which were determined empirically during preliminary experiments and subsequently fixed for all evaluated architectures. A fixed random seed of 42 was used throughout all experiments to ensure reproducibility. The final training configuration is summarized in Table 4.

All experiments were executed on a workstation running Ubuntu 24.04 LTS, equipped with an Intel Core i9-14900K CPU, 128 GB RAM, and an NVIDIA GeForce RTX 3090 Ti (24 GB) GPU. The entire framework was implemented in TensorFlow 2.14 with Keras 3, and model evaluation was performed using standard metrics including accuracy, precision, recall (sensitivity), specificity, F1-score, and the area under the ROC curve (AUC).

### 4.3. Training and Validation Results

The 10-fold cross-validation results, summarized in Table 5, show a clear and logical progression from a volatile baseline to stable, attention-regularized models. The analysis begins with the ResNet50 architecture, which serves as a performance benchmark. The baseline ResNet50 demonstrated the weakest average performance and the greatest inconsistency across the validation folds. It achieved a mean accuracy of 0.778 ± 0.039 and an F1-score of 0.764 ± 0.048. Most critically, its sensitivity was not only the lowest at 0.732 but also highly variable, with a standard deviation of 0.126 and individual fold results ranging from a high of 0.933 down to a near failure case of 0.540. This combination of moderately high precision (0.828 ± 0.091) with low, unstable sensitivity reveals a strong conservative bias; the model tends to avoid false positives at the significant cost of missing true positive cases. This behavior changed markedly depending on the data in each fold, indicating a tendency for the model to under-detect positive cases. Folds containing smaller, low-contrast adenomas or those with atypical enhancement patterns caused the model’s recall to collapse, suggesting that a purely convolutional architecture struggles to generalize subtle lesion cues among variations in patient anatomy, image noise, or phase contrast. On the other hand, the introduction of block-level attention in the Res-BLANet model led to a clear improvement in central performance metrics, though it did not uniformly resolve the issue of instability. The model achieved a higher mean accuracy of 0.825 ± 0.031 and an F1-score of 0.817 ± 0.037. Notably, the average sensitivity increased from 0.732 to 0.791, confirming that the local re-weighting of features helps the network better identify adenoma textures and phase-dependent contrast edges. However, this gain in sensitivity came with a trade-off. The model’s precision remained highly variable, with a standard deviation of approximately 0.088 and a minimum value of 0.702 in one fold. This indicates that certain data splits produced a high number of false positives. The mechanistic explanation for this behavior is that BLAM, by design, amplifies local textures. In folds that contained challenging features like vascular overlap, motion artifacts, or heterogeneous soft-tissue backgrounds, this local attention mechanism could over-respond to non-lesion high-intensity patterns, thereby increasing the false positive count. In short, while BLAM improves the model’s ability to discriminate local features, it can overfit to fold-specific texture noise when it is not guided by a broader, global context.

To provide this missing global context, we introduced stage-level attention in the Res-SLANet architecture, which led to significant improvements in both average performance and model stability. This model produced the best generalization balance, achieving a mean accuracy of 0.874 ± 0.029 and a high precision of 0.926 ± 0.067. This strategic shift led to two major changes in performance. First, the variability in sensitivity was dramatically reduced (std 0.126 in the baseline vs. 0.054 in the Res-SLANet), which eliminated the recall collapses seen in ResNet50. Second, the model maintained high precision without the outlier folds that negatively affect the performance of Res-BLANet. This is precisely what the Stage-level Light Attention Module (SLAM) is designed to achieve. By combining multi-scale context from across the network’s stages, the model becomes less sensitive to fold-specific background textures and is better able to stabilize its decision boundaries. In practice, this means that folds where the baseline ResNet50 failed due to low recall were rescued by the global anatomical context provided by SLAM. At the same time, folds where Res-BLANet produced too many false positives were tempered by this same contextual information, leading to a more balanced and reliable model.

While Res-SLANet provided the best generalization balance, the hybrid Res-LLANet architecture achieved a comparable level of performance while rebalancing the model’s bias towards maximizing recall. This model produced the most stable results of the entire study, with the lowest variance in both accuracy (0.872 ± 0.013) and F1-score (0.871 ± 0.015). It also demonstrated the strongest overall detection capability, achieving the highest mean sensitivity of 0.867 ± 0.065. However, this enhanced detection came with a modest trade-off in precision (0.881 ± 0.051), particularly on folds containing ambiguous, high-intensity structures. This behavior can be explained mechanistically. By combining both block-level and stage-level attention, Res-LLANet can retain fine-grained boundary details while using global context. This powerful combination pushes the decision threshold slightly toward the positive class, resulting in fewer false negatives and a high, stable recall across all folds. The cost of this sensitivity-focused approach is a slight increase in false positives on visually challenging vascular or noisy slices. This emphasizes an important discovery that outlier folds in one model were frequently balanced by the architecture of another. For example, folds with low recall in ResNet50 exhibited better performance when paired with Res-LLANet, whereas the high false-positive folds from Res-BLANet were normalized by the global context offered in the hybrid model.

These individual model performances show a clear pattern. The baseline ResNet50 usually underfits and struggles to capture subtle lesion cues. This leads to high variance and low recall. Res-BLANet, which uses local attention, slightly overfits to local textures. This improves recall, but it also leads to variable precision. In contrast, Res-SLANet achieves the best generalization balance between high precision and moderately high recall. Finally, Res-LLANet minimizes variance and maximizes recall, accepting a small, acceptable drop in precision to achieve the highest detection rate. Statistically, this pattern suggests that the inter-fold variability decreases as the attention mechanisms become more sophisticated. The standard deviation of the F1-score, for instance, drops from 0.048 in ResNet50 to 0.037 in Res-BLANet, to 0.027 in Res-SLANet, and finally to just 0.015 in Res-LLANet. The standard deviation of the accuracy shows a similar tightening, reaching its lowest point of 0.013 in the Res-LLANet model. This clear quantitative trend confirms the powerful stabilizing effect of hierarchical attention on the model’s performance across different patient data splits.

This balancing effect can be explained by how the different attention mechanisms interact with the specific composition of the data within each fold. Several key factors influenced this fold-level behavior. For instance, folds enriched with small or low-contrast adenomas caused a drop in recall for the ResNet50 baseline. These challenging cases were successfully identified by the local feature enhancement of BLAM and LLANet, while the global context provided by SLAM also contributed to their correct classification. Conversely, folds that included images with significant vascular overlap or streak artifacts led to an increase in false positives for Res-BLANet, as its local attention mechanism incorrectly amplified these non-lesion features. In these cases, SLAM’s contextual gating was able to reduce the false positive rate. Furthermore, variations in contrast phase timing or Hounsfield Unit shifts could destabilize the non-attention models, but the attention modules indicated more robust by re-centering the model’s focus on consistent gland boundaries. Finally, in folds with fewer positive cases, the ResNet50 baseline developed a negative bias, increasing precision at the cost of recall. However, the Res-LLANet maintained its high sensitivity due to its inherent recall bias, even when there were few positive examples. These interactions explain the migration of outlier performance between models. For example, folds where the baseline model missed detections were often corrected by the enhanced feature focus of Res-LLANet. In contrast, folds where Res-BLANet had poor precision were improved by the contextual regularization provided by SLAM.

### 4.4. Training and Validation Plots for the Best Folds

**Figure 12:**
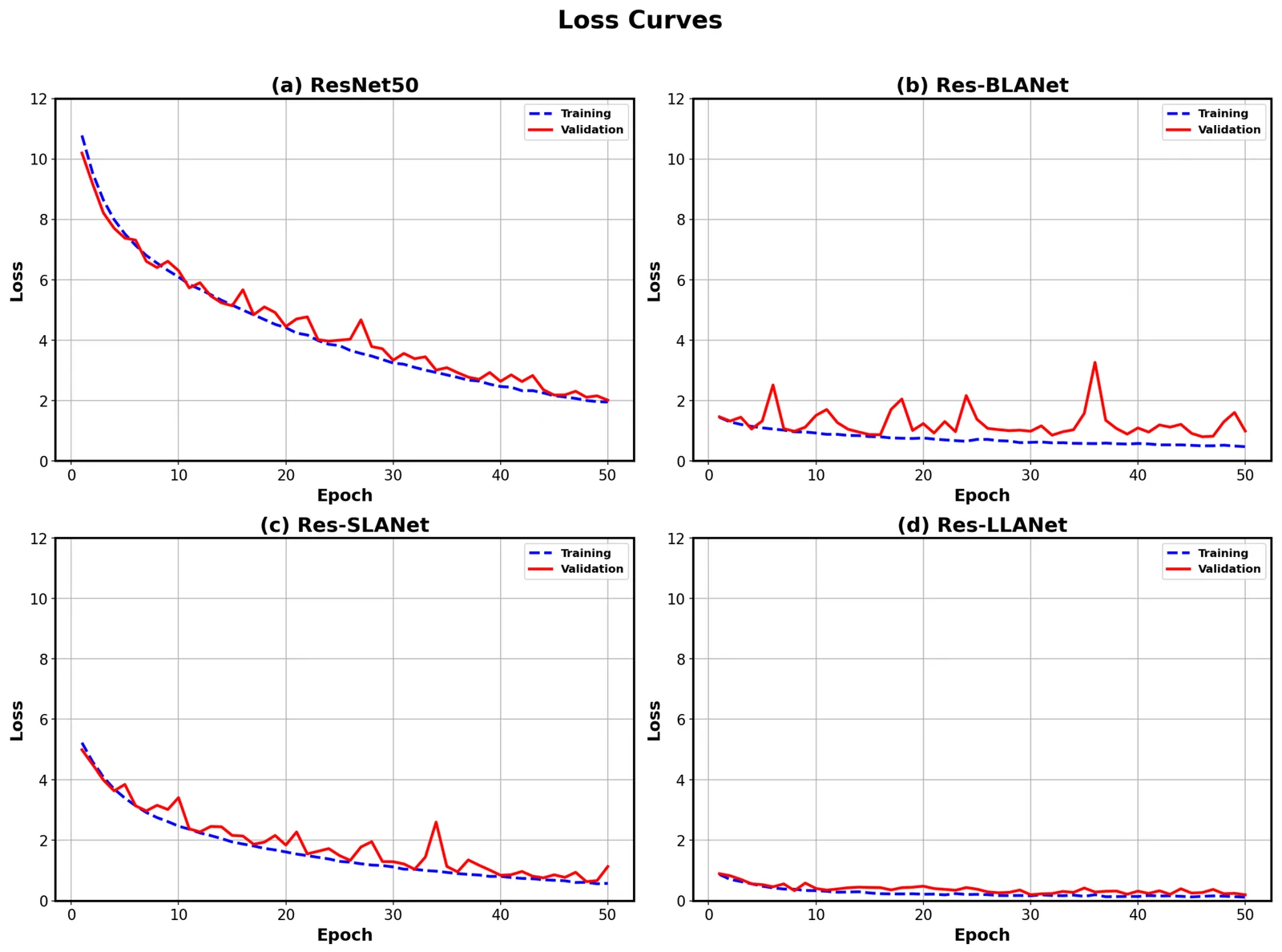
Comparison of Loss in training and validation of models.

**Figure 13:**
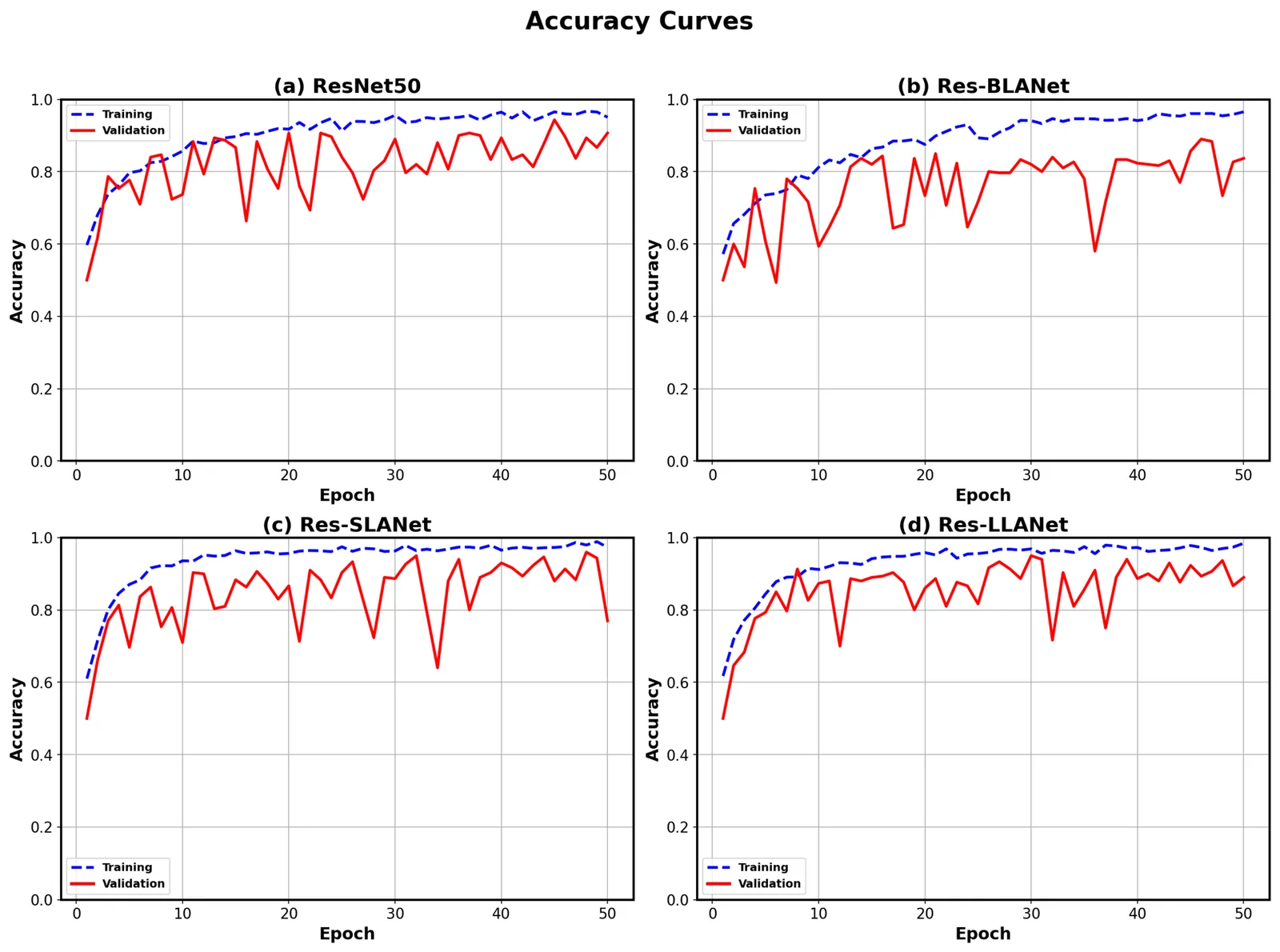
Comparison of Accuracy in training and validation of models.

**Figure 14:**
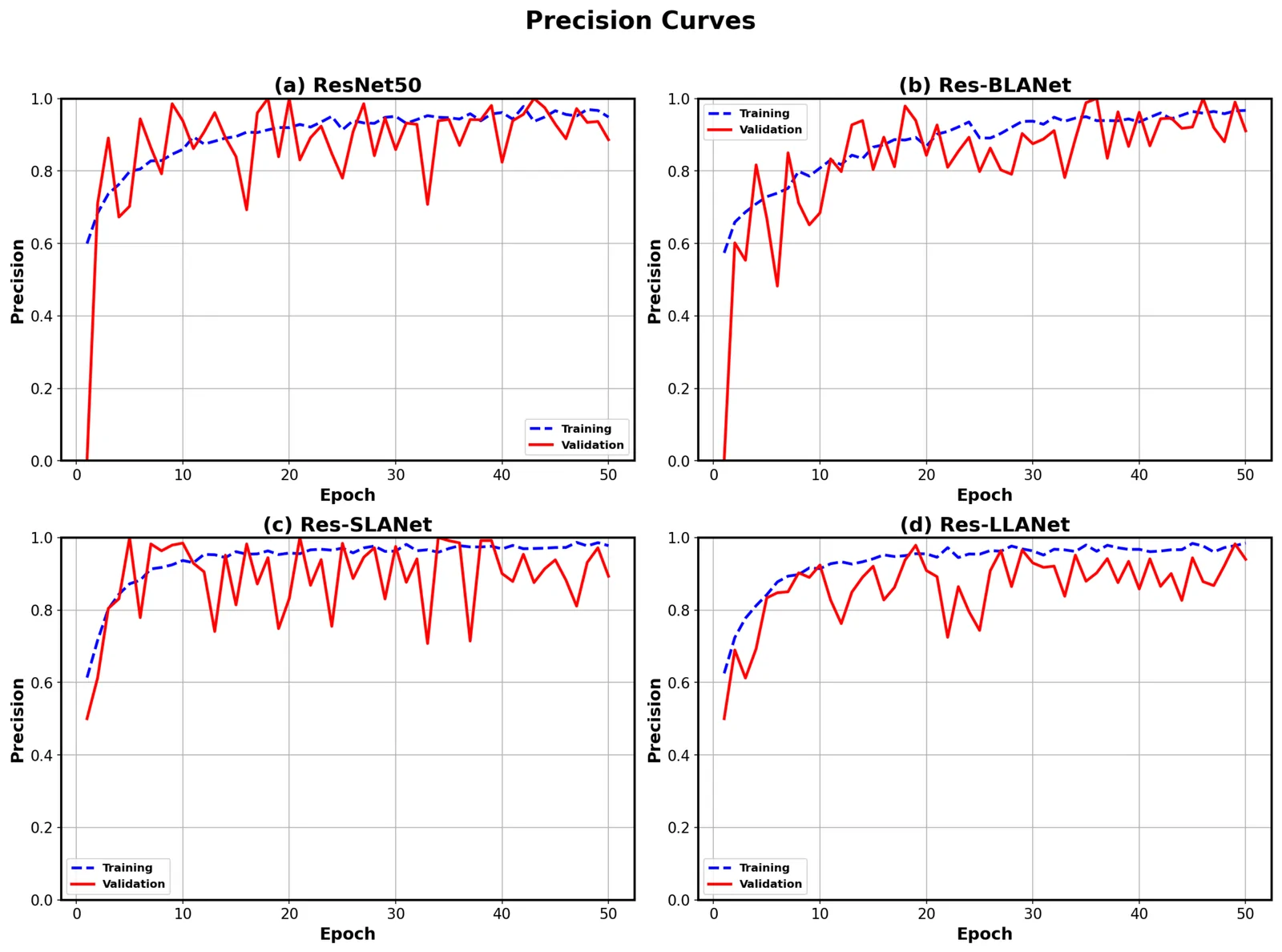
Comparison of Precision in training and validation of models.

**Figure 15:**
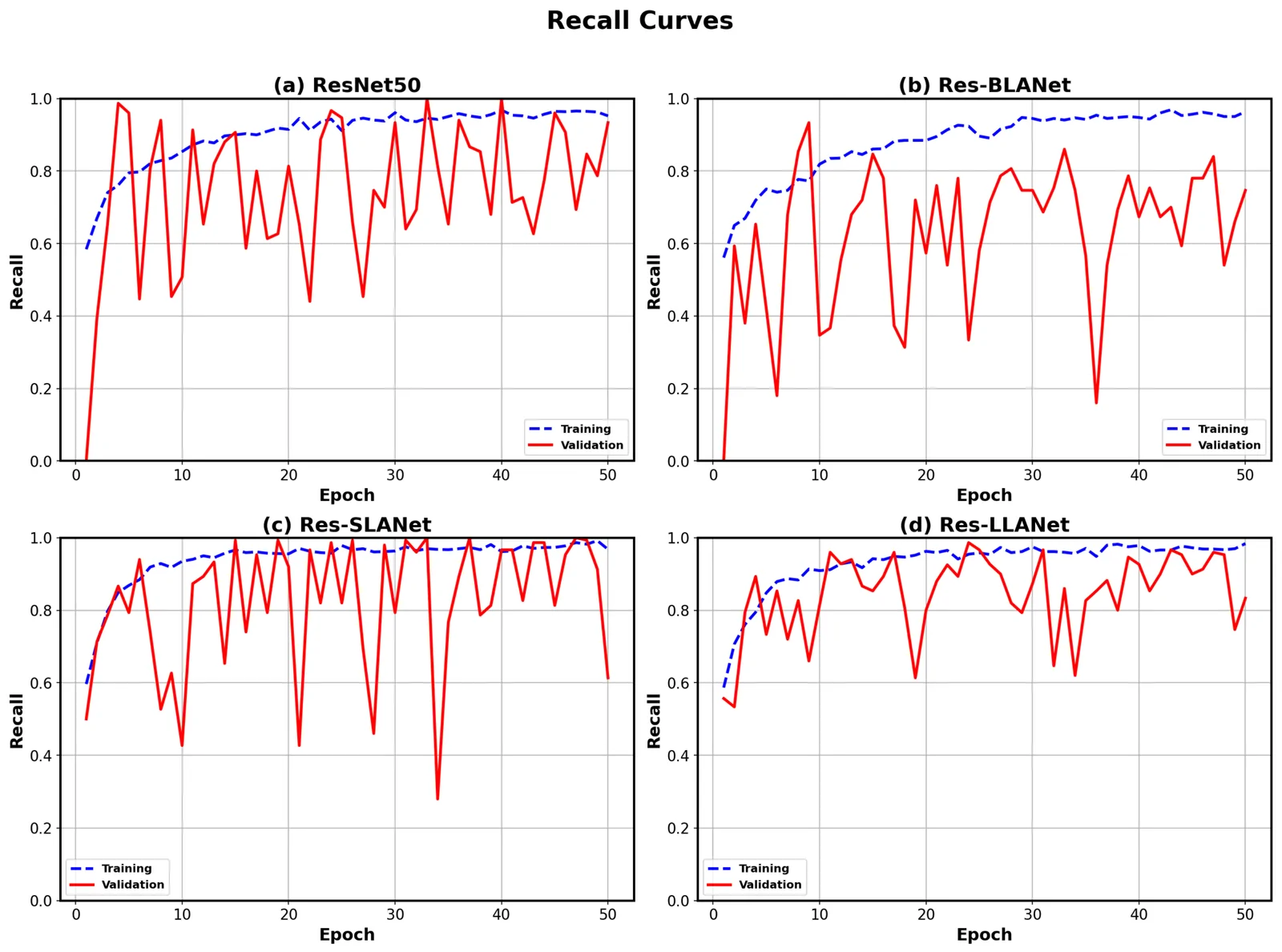
Comparison of Recall in training and validation of models.

To qualitatively assess the learning dynamics and stability of each model, we analyzed the performance plots for their respective best-performing validation folds over 50 training epochs. The plots for loss, accuracy, precision, and recall, shown in Figure 12, Figure 13, Figure 14 and Figure 15 respectively, provide a visual comparison of how each architecture learned and generalized over time. The loss curves shown in Figure 12 highlight clear differences in model performance. The baseline ResNet50 consistently demonstrated the highest validation loss throughout the training process, indicating weaker generalization compared to the other models. In contrast, the attention-based models all achieved lower validation losses. In particular, the Res-LLANet exhibited a smooth and steady decay, reaching the lowest validation loss among all models and demonstrating a superior ability to generalize from the training data. Figure 13 illustrates the training and validation accuracy plots. While all models show a steep rise in accuracy during the initial epochs and stable convergence on the training data, the Res-BLANet model consistently reached the lowest training accuracy among the group. In contrast, its validation accuracy was highly variable throughout the training process, indicating potential instability in its ability to generalize. The validation curves for ResNet50 and Res-SLANet demonstrated comparatively fewer fluctuations, reflecting a more reliable learning behavior. Notably, the Res-LLANet model achieved the most consistent and steady validation accuracy with minimal oscillations, suggesting it possesses superior generalization and robustness among the evaluated architectures. Figure 14 illustrates the precision performance of the four models. After a rapid improvement during the initial epochs, all architectures demonstrate smooth and stable convergence in both their training and validation precision. This result suggests that all models, including the baseline, are effective at minimizing false positives and can maintain consistent predictive reliability from a precision standpoint. In contrast, the recall trends in Figure 15 show significant variability in the models’ ability to detect true positive cases. While the training recall curves for all architectures converge steadily, the validation recall for ResNet50, Res-BLANet, and Res-SLANet exhibits pronounced fluctuations throughout the training process. This indicates a persistent instability in their capacity to capture all true positive instances. The Res-LLANet model, however, stands out by demonstrating the most stable recall behavior. It maintains a high validation recall with minimal fluctuations from the early epochs, highlighting its superior generalization in this critical, sensitivity-related metric. Collectively, the plots clearly show that while the ResNet50 baseline struggled with unstable learning, the introduction of attention progressively improved the training dynamics. The Res-SLANet and Res-LLANet models, in particular, demonstrated the most balanced and stable behavior across all metrics, showing consistent generalization from the training data. This visual analysis reinforces the conclusion that these two architectures are the most robust and are the best candidates for further testing and implementation.

### 4.5. Models Inference Results

**Figure 16:**
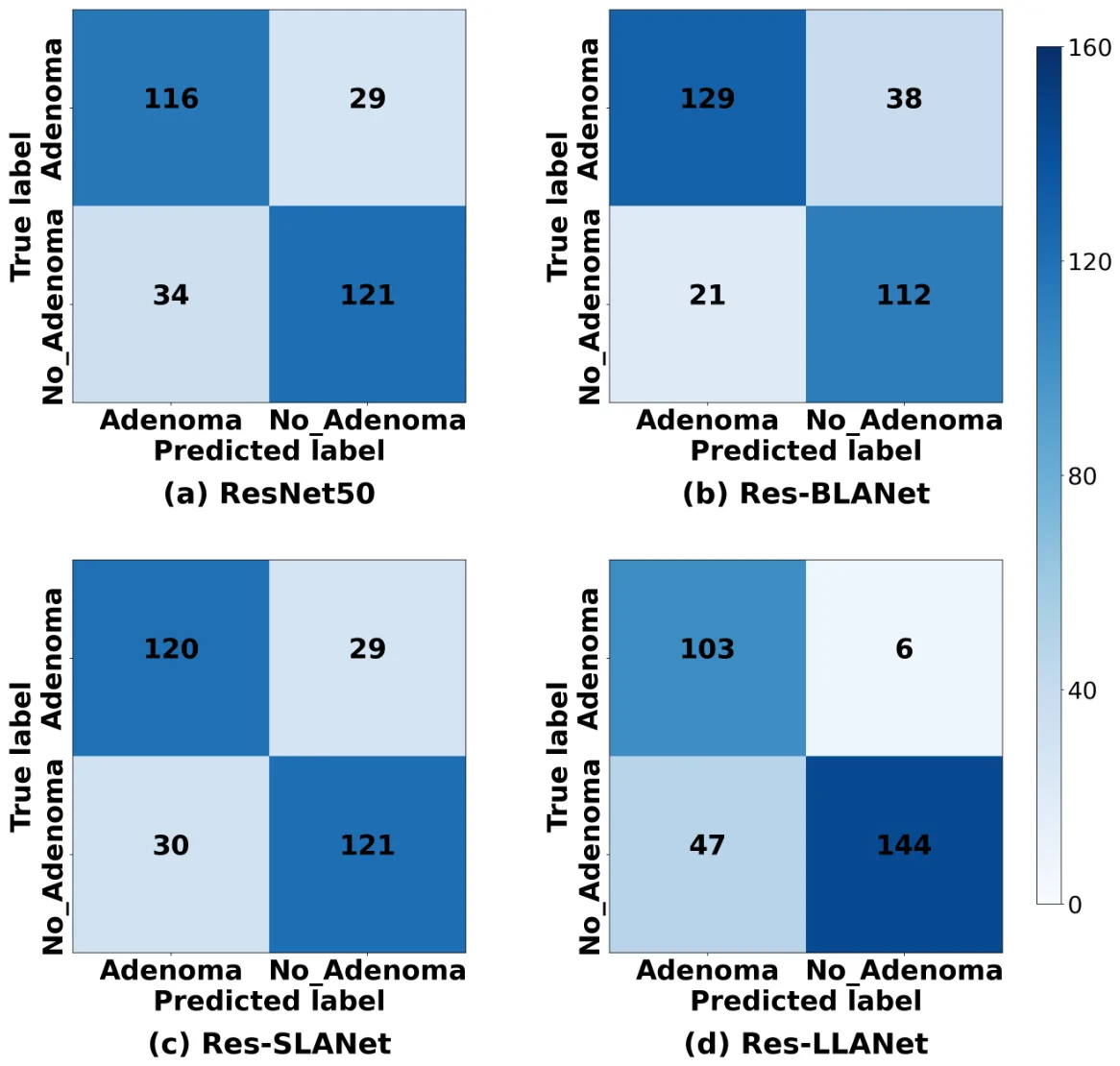
Comparison of confusion matrices for the baseline ResNet50 model and the proposed Res-BLANet, Res-SLANet, and Res-LLANet architectures evaluated on the unseen test dataset.

**Figure 17:**
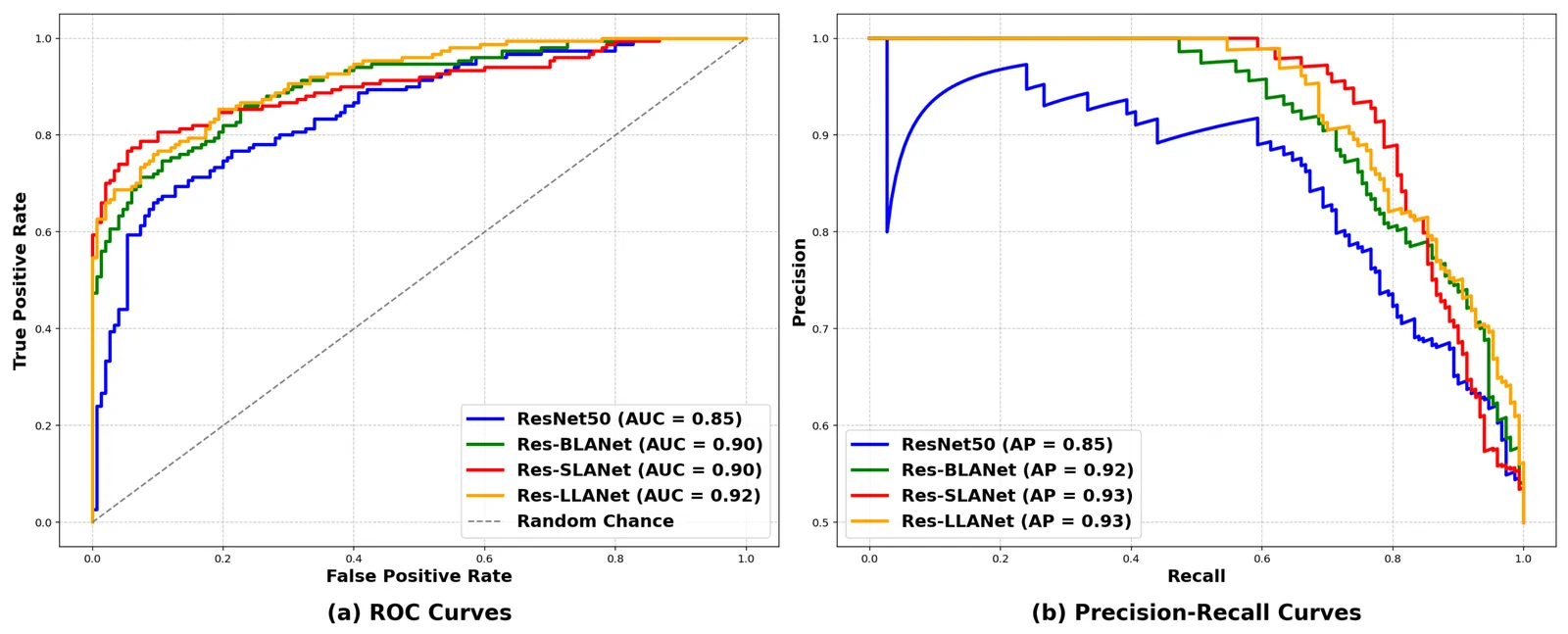
Comparison of ROC and PR Curves for the baseline ResNet50 model and the proposed Res-BLANet, Res-SLANet, and Res-LLANet architectures evaluated on the unseen test dataset.

Table 6 summarizes the performance of the proposed models on the independent testing dataset with their corresponding 95% confidence intervals. Figure 16 illustrates the corresponding confusion matrices. True Positives (TP), True Negatives (TN), False Positives (FP), False Negatives (FN), Accuracy, Precision, Sensitivity (Recall), and F1-score were among the performance metrics used to evaluate the models, whereas, Figure 17 presents the ROC and precision–recall curves for visual comparison of the classification performance. Together, these measurements provide a comprehensive view of diagnostic dependability by capturing the trade-offs between sensitivity and specificity.

Res-LLANet outperformed all other studied architectures, properly detecting nearly all positive cases while limiting false negatives (FN = 6). Its overall accuracy was 82.3%, while its sensitivity was the greatest at 96.0%. This is especially crucial in clinical diagnosis since misleading negative results might result in pathological problems going undiagnosed and medical care being ignored. The higher number of false positives (FP = 47) results in a somewhat lower precision (0.753), but the F1-score of 0.844 show relatively good predictive reliability and agreement beyond chance.

With an accuracy of 80.3% each, the Res-BLANet and Res-SLANet variants demonstrated balanced performance. When too many false positives could result in needless diagnostic procedures, Res-BLANet’s precision (0.842) was the greatest of all the models, demonstrating its efficacy in lowering false alarms. The very low sensitivity (0.746), however, suggests a slightly increased chance of missed detections. Res-SLANet, on the other hand, provided a steady compromise between reducing false positives and false negatives by providing a balanced trade-off between precision (0.801) and sensitivity (0.806).

The baseline ResNet50 performed the worst in comparison, with an accuracy of 79.0% and an F1-score of 79.3%. These findings demonstrate that the proposed lightweight attention mechanisms effectively enhance feature representation and contextual learning, leading to improved classification performance. Res-LLANet obtained the largest Area Under the Curve (AUC = 0.92), whereas ResNet50 obtained the lowest (AUC = 0.85), according to the ROC Curves. According to the Precision-Recall Curves, Res-LLANet had the highest Average Precision (AP = 0.93), closely followed by Res-SLANet and Res-BLANet (AP = 0.93 and 0.92, respectively). These gains in AUC and AP scores attest to the efficiency of multi-level light attention modules in identifying discriminative characteristics and eliminating superfluous noise from the data.

The findings carry significant clinical implications. A diagnostic model with high sensitivity, such as Res-LLANet, ensures that nearly all patients with parathyroid adenoma are correctly identified, minimizing the risk of missed diagnoses. This is critical since delayed or missed detection can lead to complications such as persistent hyperparathyroidism, bone demineralization, and renal impairment. Although the model shows a moderate rate of false positives, in a real-world clinical workflow, such cases would typically undergo further imaging or biochemical tests, making a sensitivity-prioritized model preferable over one that sacrifices true positive detection for fewer false alarms.

It should be emphasized that the proposed framework performs binary classification at the slice level rather than direct patient-level diagnosis. This distinction is important because slice-level classification evaluates the model’s ability to recognize imaging features associated with parathyroid adenoma, whereas patient-level diagnosis requires aggregating information across all slices belonging to an individual examination. In clinical practice, predictions from all arterial-phase CT slices belonging to the same patient would be aggregated to generate a single patient-level diagnosis. In the present study, this aggregation was performed by averaging the predicted probabilities across all slices belonging to an individual patient and assigning the final diagnosis using a classification threshold of 0.5. Although this simple probability-averaging strategy provides an initial estimate of patient-level performance, it should be regarded as a preliminary proof-of-concept because of the limited size of the independent test split.

To complement the slice-level evaluation, patient-level predictions were generated for the independent test set using the aggregation strategy described above. The patient-level results are summarized in Table 7. Of the three independent patients, one patient with parathyroid adenoma (Patient 1) and the negative patient (Patient 3) were correctly classified, whereas one adenoma patient (Patient 2) was incorrectly classified as negative. Consequently, the proposed framework correctly identified two of the three independent patients, corresponding to a patient-level accuracy of 66.7%, a patient-level sensitivity of 50.0% (1/2), and a specificity of 100% (1/1) (Table 8). Although these patient-level results provide an initial indication of the model’s ability to generalize to previously unseen patients, the extremely limited size of the independent test split prevents robust estimation of clinical performance. Accordingly, the reported 95% confidence intervals were estimated using bootstrap resampling; however, due to the inclusion of only three independent patients, these intervals are inherently wide and should be interpreted with caution, as they primarily reflect the uncertainty associated with the small sample size rather than the true variability of model performance. Therefore, the patient-level findings should be regarded as preliminary evidence and require validation on substantially larger, independent external cohorts before definitive conclusions regarding clinical generalizability can be drawn.

### 4.6. Diagnostic Performance, Model Complexity and Computational Cost

The comprehensive evaluation of the proposed architectures, which encompasses both the diagnostic performance on the testing dataset (Table 6) and the computational efficiency (Table 9), reveals a crucial trade-off that defines the potential for clinical deployment of these architectures. Despite the fact that the baseline ResNet50 model provides a performance that is balanced but moderate (Accuracy: 0.790, F1: 0.793), each of our proposed models excels in significant areas that are distinct from one another. Res-BLANet emerges as the most efficient option, offering an inference speed that is three times quicker than ResNet50 (24.44 frames per second against 8.06 frames per second), making it the most promising alternative for real-time applications. Although it has a little lower sensitivity (0.746), this efficiency comes with a high precision of 0.842, which indicates that it is good at minimising false positives. However, this comes at the expense of higher precision. On the other hand, Res-LLANet exhibits remarkable diagnostic capacity, achieving the highest sensitivity (0.960) and F1-score (0.844), which indicates that it is unrivalled in its ability to correctly identify all genuine cases and provide the most reliable overall predictions. The slowest inference speed (8.90 frames per second) and the longest training time are also associated with this high accuracy, which comes at a large cost to the computational resources. The Res-SLANet offers a balanced improvement over the baseline in terms of both performance (Accuracy: 0.803) and efficiency. Res-SLANet achieves a balanced compromise between predictive performance and computational efficiency. Therefore, the choice of architecture should be guided by the intended clinical application. Res-BLANet is particularly suitable for high-throughput screening environments where fast inference is essential. Conversely, Res-LLANet is better suited for diagnostic settings that demand the highest possible detection performance, owing to its superior sensitivity and overall classification accuracy. Res-SLANet provides a versatile alternative, offering a favorable balance between accuracy, robustness, and computational cost for routine clinical decision-support systems.

## 5. Statistical Analysis

To determine whether statistically significant differences existed among the evaluated models (ResNet50, Res-BLANet, Res-SLANet, and Res-LLANet), a non-parametric statistical analysis was performed using the fold-wise F1-scores obtained from the 10-fold cross-validation. Each cross-validation fold was treated as one paired observation, resulting in ten paired observations for each model. The Friedman test was first employed as an omnibus test to compare the performance of all models simultaneously. The null hypothesis (H_0_) states that there is no significant difference in the F1-score distributions among the evaluated models, whereas the alternative hypothesis (H_1_) states that at least one model performs significantly differently.

Table 10 summarizes the descriptive statistics of the fold-wise F1-scores for all evaluated models. Res-LLANet achieved the highest mean F1-score (0.8707) while exhibiting the smallest standard deviation (0.0148), indicating not only superior classification performance but also the greatest consistency across the ten cross-validation folds. Res-SLANet ranked second with a mean F1-score of 0.8669, followed by Res-BLANet (0.8169), whereas the baseline ResNet50 achieved the lowest mean performance (0.7642) and the largest performance variability. These descriptive statistics suggest that progressively incorporating lightweight attention mechanisms improves both classification accuracy and stability.

**Figure 18:**
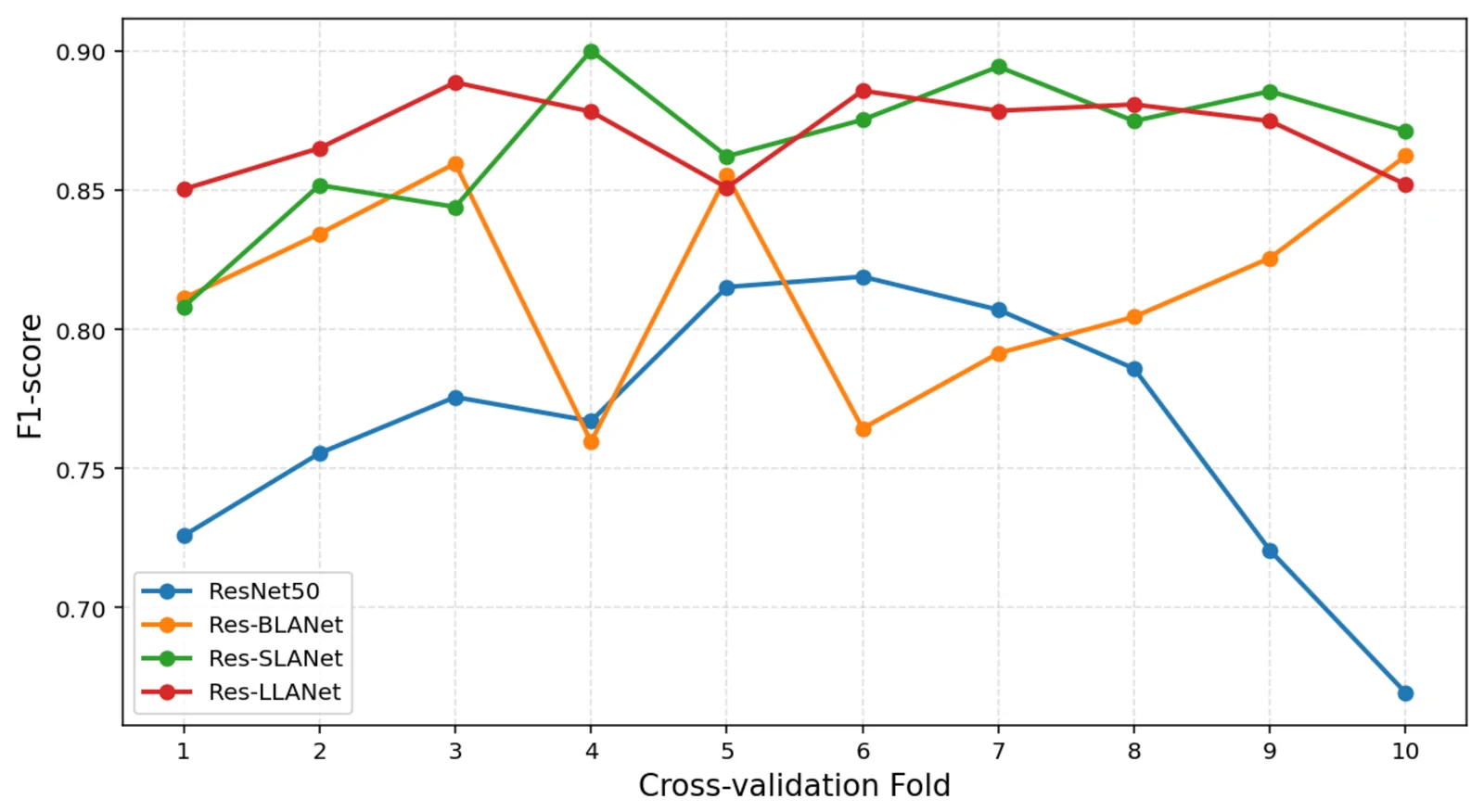
Fold-wise F1-scores of the evaluated models across the stratified 10-fold cross-validation.

Figure 18 illustrates the fold-wise F1-score obtained by each model during the 10-fold cross-validation. The proposed Res-LLANet consistently achieved the highest or near-highest F1-score across almost every fold, demonstrating stable performance and superior generalization. Res-SLANet also exhibited competitive performance but showed slightly larger variations between folds. In contrast, ResNet50 demonstrated both lower F1-scores and greater variability, indicating reduced robustness. The consistency observed for Res-LLANet visually supports the statistical analysis presented in the following sections.

The Friedman test (Table 11) revealed a statistically significant overall difference among the four evaluated models ($\chi^{2}=17.280$, p=0.000619). Consequently, the null hypothesis was rejected, indicating that the observed differences in fold-wise F1-scores were unlikely to have occurred by chance.

Following the Friedman test, post hoc pairwise comparisons were performed using the Wilcoxon signed-rank test (Table 12). Since multiple pairwise comparisons were conducted, the Holm–Bonferroni correction was applied to control the family-wise error rate. The corrected results demonstrated that all pairwise comparisons remained statistically significant ($p_{\mathrm{adj}}<0.05$). Specifically, Res-BLANet significantly outperformed the baseline ResNet50 ($p_{\mathrm{adj}}=0.0098$), while Res-SLANet significantly outperformed both ResNet50 ($p_{\mathrm{adj}}=0.0117$) and Res-BLANet ($p_{\mathrm{adj}}=0.0059$). Furthermore, the proposed Res-LLANet achieved statistically significant improvements over ResNet50 ($p_{\mathrm{adj}}=0.0117$), Res-BLANet ($p_{\mathrm{adj}}=0.0391$), and Res-SLANet ($p_{\mathrm{adj}}=0.0054$).

Overall, the descriptive statistics, fold-wise performance trends, and non-parametric statistical analyses consistently demonstrate a clear performance hierarchy among the evaluated architectures. The gradual incorporation of lightweight attention mechanisms resulted in progressive improvements over the baseline ResNet50. Among all evaluated models, Res-LLANet achieved the highest mean fold-wise F1-score together with the lowest performance variability, indicating both superior classification accuracy and excellent generalization across the cross-validation folds. Furthermore, the Friedman test confirmed a significant overall difference among the models, while the post hoc Wilcoxon signed-rank tests with Holm–Bonferroni correction demonstrated that the improvements achieved by Res-LLANet over all competing architectures were statistically significant. These findings provide strong evidence for the effectiveness and robustness of the proposed lightweight local attention mechanism for parathyroid adenoma classification.

## 6. Explainable Artificial Intelligence (XAI) Analysis

To provide qualitative insight into the prediction process of the proposed model, we analyzed the attention regions using Gradient-weighted Class Activation Mapping (Grad-CAM). We have selected the Res-LLANet model, which has both the characteristics of SLANet and BLANet. To investigate whether the network focuses on clinically relevant image regions, Gradient-weighted Class Activation Mapping (Grad-CAM) was employed. Grad-CAM provides visual explanations by highlighting spatial regions within the input image that contribute most strongly to the model’s prediction.

For each CT slice, Grad-CAM maps were generated using the gradients flowing into the final convolutional layer of the network. These gradients were used to compute importance weights for the feature maps, which were then combined to produce a coarse localization heatmap. The resulting activation maps were upsampled and overlaid on the original CT images to visually indicate the regions influencing the classification decision.

**Figure 19:**
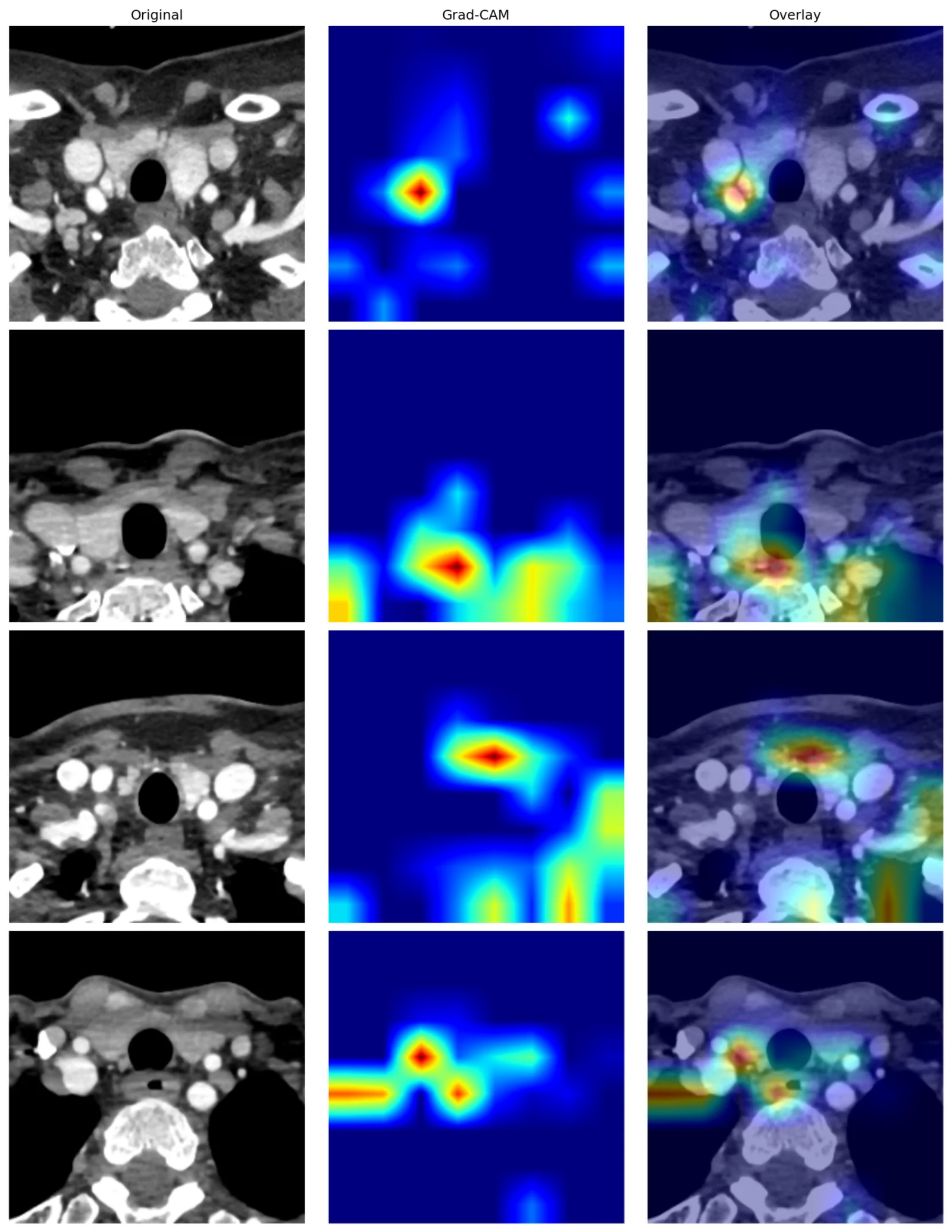
Grad-CAM–based explainability of the Res-LLANet model for parathyroid adenoma detection on axial CT slices. CT Slices of correctly classified cases are shown. Each row corresponds to a different patient slice and contains three panels: (left) the original axial CT image of the neck, (middle) the Grad-CAM heatmap indicating regions contributing most strongly to the model’s prediction, and (right) the overlay of the heatmap on the original CT slice. Warmer colors (red/yellow) represent regions with a higher contribution to the classification decision, whereas cooler colors (blue) indicate low relevance. The highlighted regions correspond to focal areas adjacent to the thyroid gland where parathyroid adenomas are typically located, suggesting that the proposed Res-LLANet model focuses on clinically relevant anatomical structures during prediction.

Figure 19 presents representative examples of Grad-CAM visualizations for correctly classified cases of parathyroid adenoma. Each example contains three panels: the original CT slice, the Grad-CAM activation heatmap, and the overlay visualization combining both. Warmer colors (red/yellow) indicate regions that strongly influenced the model’s prediction, while cooler colors (blue) indicate low contribution.

Across multiple samples, the activation maps consistently highlight focal regions adjacent to the thyroid gland where parathyroid adenomas are typically located. The activation maps generally concentrate on small nodular structures in the expected anatomical region rather than diffuse background areas, suggesting that the model has learned clinically relevant spatial features. The overlay visualizations qualitatively demonstrate that the highest activations correspond closely to the suspected adenomatous tissue visible in the CT slices.

The Grad-CAM visualizations suggest that Res-LLANet primarily relies on localized image regions rather than broadly distributed background information when performing classification. Instead, the model predominantly focuses on localized areas corresponding to the suspected adenoma. This observation aligns with radiological interpretation patterns, where radiologists identify parathyroid adenomas based on focal soft-tissue nodules adjacent to the thyroid or in the tracheoesophageal groove.

Moreover, the activation maps show that the model captures both structural and contextual cues, including surrounding vascular structures and tissue boundaries that may aid in differentiating adenomatous tissue from normal anatomy. These visualizations provide qualitative evidence that the learned features correspond to medically meaningful image characteristics.

## 7. Ablation Study of Hierarchical Attention Components

To further investigate the contribution of the proposed architectural components, a progressive ablation study was performed by evaluating four representative network variants, as summarized in Table 13. Since BLAM and SLAM are structurally integrated within the proposed residual framework rather than designed as independent plug-in attention modules, the ablation study was conducted at the architecture level instead of disabling individual components. Specifically, ResNet50 serves as the baseline backbone without any attention mechanism. Res-BLANet evaluates the contribution of the proposed block-level attention by integrating BLAM within the proposed Hybrid Bottleneck Attention (HBA) and Hybrid Identity Attention (HIA) residual blocks. Res-SLANet investigates the effectiveness of the stage-level attention mechanism by incorporating SLAM after each residual stage. Finally, Res-LLANet combines both attention mechanisms through the proposed hierarchical feature fusion strategy, allowing complementary local and global contextual representations to be jointly exploited for classification.

Compared with the baseline ResNet50, Res-BLANet consistently improves the classification performance, demonstrating that incorporating block-level attention within the residual blocks enables the network to better suppress irrelevant background information while enhancing local discriminative features associated with parathyroid adenomas.

Res-SLANet also outperforms the baseline by introducing stage-level attention that aggregates contextual information across an entire residual stage. This indicates that modeling long-range contextual relationships further improves feature representation beyond purely local attention.

The proposed Res-LLANet achieves the best overall performance by integrating both BLAM and SLAM through the proposed hierarchical feature fusion mechanism. The fusion strategy combines residual features with their corresponding attention-enhanced representations, allowing local discriminative information extracted by BLAM to complement the global contextual representations generated by SLAM. The superior performance of Res-LLANet demonstrates that the improvements arise from the complementary interaction between local and stage-level attention rather than from either attention mechanism individually.

It should be noted that BLAM, SLAM, HBA, HIA, and the attention–residual feature fusion mechanism are not independent architectural components but are designed as mutually dependent elements of the proposed framework. In each proposed architecture, the attention maps are first aggregated and subsequently fused with the corresponding residual feature maps before classification. Consequently, BLAM and SLAM cannot be evaluated independently of the proposed feature fusion strategy because the fusion operation constitutes an integral part of the network design. Therefore, ablation at the architecture level provides a more meaningful evaluation of the individual architectural contributions than removing these components independently, which would fundamentally alter the proposed network topology.

## 8. Discussion

The results of this study demonstrate that the proposed hierarchical attention-based architectures achieve competitive performance for parathyroid adenoma detection from arterial-phase CT imaging while simultaneously providing computational efficiency and qualitative model interpretability. As summarized in Table 1, recent artificial intelligence approaches for parathyroid localization and classification have reported strong diagnostic performance across multiple imaging modalities, including scintigraphy, SPECT/CT, 4D-CT, PET, and near-infrared autofluorescence imaging.

Among the proposed models, Res-LLANet achieved the highest sensitivity while maintaining competitive overall performance. Although several previous studies reported higher accuracy values, particularly those based on scintigraphy datasets [11,12,13], direct comparison should be interpreted cautiously because of substantial differences in imaging modality, dataset characteristics, patient populations, and evaluation protocols. Nevertheless, the sensitivity achieved by Res-LLANet is comparable to, and in some cases exceeds, that reported by several established deep learning approaches. From a clinical perspective, high sensitivity is particularly important because missed adenomas may lead to delayed diagnosis and treatment.

To further evaluate the effectiveness of the proposed architectures, we compared them with several widely adopted convolutional neural network architectures, including ConvNeXt-Base, DenseNet201, EfficientNetV2L, VGG19, InceptionV3, Xception, and ResNet50, all trained from scratch using identical training configurations and random weight initialization. As shown in Table 14, the proposed attention-enhanced residual architectures consistently achieved superior performance compared with the conventional baseline models. Among the reference architectures, ResNet50 achieved the strongest overall performance with an accuracy of 79.0% and an F1-score of 79.3%, followed by Xception with an accuracy of 77.33% and an F1-score of 76.55%. In contrast, ConvNeXt-Base, DenseNet201, EfficientNetV2L, VGG19, and InceptionV3 exhibited comparatively lower classification performance, achieving F1-scores ranging from 59.73% to 71.45%. The proposed Res-BLANet further improved precision to 84.2%, demonstrating that the integration of the Block-Level Light Attention Module effectively suppresses irrelevant background information while enhancing discriminative local features. Res-SLANet achieved a balanced improvement across all evaluation metrics, obtaining an accuracy of 80.3%, sensitivity of 80.6%, and an F1-score of 80.3%, indicating that stage-level contextual attention enhances feature representation throughout the network. Among all evaluated models, Res-LLANet demonstrated the best overall performance, achieving the highest accuracy (82.3%), sensitivity (96.0%), and F1-score (84.4%). The substantial improvement in sensitivity is particularly important for clinical applications, as it reduces the likelihood of missed parathyroid adenoma slices while maintaining competitive precision. Overall, these findings suggest that incorporating lightweight hierarchical attention mechanisms within the residual learning framework yields more discriminative feature representations than conventional convolutional architectures trained under identical conditions, thereby improving slice-level classification performance without introducing excessive computational complexity.

An important contribution of this work is the investigation of the trade-off between diagnostic performance and computational efficiency. While many existing studies focus primarily on predictive accuracy, limited attention has been given to computational requirements and real-time deployment considerations. For example, transformer-based architectures have demonstrated strong performance in medical imaging tasks but are often associated with increased computational complexity and inference costs. In contrast, the proposed Res-BLANet architecture was designed with efficiency as a primary objective. Despite a modest reduction in sensitivity compared with Res-LLANet, Res-BLANet achieved high precision and substantially faster inference speed, making it attractive for integration into clinical workflows where rapid image analysis is required.

Another distinguishing aspect of the proposed framework is its emphasis on qualitative model interpretability. Interpretable AI systems have become increasingly important in medical imaging, where clinical adoption depends not only on predictive performance but also on providing visual evidence that may assist interpretation of model predictions. Previous studies have highlighted this need by incorporating explainability techniques such as Grad-CAM++ or interpretable machine learning models [10,45]. Similarly, the hierarchical attention mechanisms incorporated into Res-BLANet, Res-SLANet, and Res-LLANet generate attention maps that highlight image regions contributing to the classification decision. These visualizations provide qualitative exploratory evidence of the network’s prediction process and may assist interpretation of the model outputs.

Taken together, the improvements in diagnostic performance, computational efficiency, and qualitative interpretability highlight the potential of hierarchical attention mechanisms for parathyroid adenoma detection. The findings of this study further support the growing body of evidence demonstrating the effectiveness of deep learning for parathyroid pathology detection. Consistent with previous investigations across diverse imaging modalities [17,19,40,42,44], our results show that AI-based approaches can achieve meaningful diagnostic performance. Furthermore, the evaluation of multiple performance metrics, including accuracy, precision, sensitivity, F1-score, provides a comprehensive assessment of model behavior and offers a more balanced evaluation than reliance on a single performance measure.

Although the proposed architectures demonstrated consistent performance across the 10-fold cross-validation experiments and the independent patient-level test set, the relatively limited number of available patients and the use of a single-institution dataset warrant cautious interpretation of the reported results. Small medical imaging datasets inherently increase the risk of overfitting, particularly when deep learning models are capable of learning patient-specific imaging characteristics rather than disease-relevant anatomical features. To mitigate this risk, several complementary strategies were incorporated throughout model development. First, all dataset partitions were performed at the patient level, ensuring that slices from the same patient were never shared across the training, validation, or testing subsets, thereby preventing data leakage and artificially inflated performance. Second, 10-fold cross-validation was employed to evaluate the stability of model performance across different patient partitions rather than relying on a single train-test split. Third, offline data augmentation was applied exclusively to the training data to increase anatomical variability while preserving clinically meaningful structures, reducing the likelihood that the networks memorized specific imaging appearances. In addition, dropout regularization, L2 weight regularization, and model selection based on the highest validation F1-score were used to further improve generalization and reduce overfitting during optimization. Importantly, the final evaluation was conducted on an independent patient-level test set that remained completely unseen during training, validation, and hyperparameter tuning, providing an unbiased assessment of model performance on previously unseen patients. The comparable performance observed across the cross-validation folds and the independent test set suggests that the proposed architectures learned discriminative imaging patterns rather than simply memorizing the training data. Nevertheless, because all images originated from a single institution using a relatively homogeneous acquisition protocol, the models may still be sensitive to domain shifts arising from different scanners, imaging protocols, patient demographics, or clinical populations. Consequently, although the present findings provide encouraging evidence of robustness within the available dataset, external validation on larger multicenter dataset acquired from multiple institutions is essential before the proposed framework can be considered clinically generalizable.

Although the proposed framework performs classification at the individual slice level, it is intended to function as a component of a comprehensive computer-aided diagnosis system for parathyroid adenoma detection. In a practical clinical workflow, a complete 4D-CT examination would first undergo standard preprocessing, and the arterial phase would be extracted for analysis. The proposed model would then classify each arterial-phase CT slice independently, producing probability scores that indicate the likelihood of parathyroid adenoma presence. These slice-level predictions could subsequently be aggregated across the entire CT volume using patient-level decision strategies, such as majority voting, probability averaging, or three-dimensional spatial consistency analysis, to determine the overall diagnostic outcome. The slices identified as suspicious could then be highlighted for radiologist review, allowing rapid localization of potential adenomas while preserving physician oversight. This information could support minimally invasive surgical planning by directing attention to anatomically relevant regions before surgery. Since the present work focuses on validating the proposed attention-guided slice-level classification framework, the development of an integrated patient-level system incorporating volumetric analysis, lesion localization, and automated clinical decision support represents an important direction for future research.

## 9. Limitation

Despite these encouraging findings, several limitations should be acknowledged. First, the test set consisted of only three previously unseen patients because of the limited availability of pathologically confirmed arterial-phase CT examinations meeting the study inclusion criteria. Although patient-level data splitting prevented information leakage between training and testing, the limited number of independent patients restricts the statistical confidence with which clinical generalization can be assessed. Furthermore, the proposed framework performs slice-level classification rather than direct patient-level diagnosis, and multiple CT slices from the same patient should not be regarded as statistically independent clinical observations. Consequently, the reported results should be interpreted as preliminary evidence of the effectiveness of the proposed framework.

In addition, although Grad-CAM and the proposed hierarchical attention mechanisms consistently highlighted regions corresponding to the suspected adenoma locations, the present study did not perform quantitative localization validation using radiologist annotations (e.g., overlap metrics, pointing-game accuracy, or lesion-region attention concentration). Consequently, the attention visualizations should be interpreted as qualitative exploratory evidence rather than validated clinical explainability.

Another limitation of this study is the clinically guided slice-selection strategy. Slice extraction was restricted to the lower cervical region based on expert radiologist recommendations and the observed anatomical distribution of parathyroid adenomas within our dataset. Although this approach ensured that both positive and negative samples originated from comparable anatomical regions, it cannot completely exclude the possibility that residual positional information contributed to model performance. Future studies will evaluate the proposed framework using full CT volumes and larger multicenter datasets to further investigate the influence of sampling strategy on model generalization.

## 10. Conclusions

This study proposed three lightweight hierarchical attention-based architectures, namely Res-BLANet, Res-SLANet, and Res-LLANet, for automated slice-level detection of parathyroid adenoma from arterial-phase CT images. The proposed Block-Level Attention Module (BLAM) and Stage-Level Attention Module (SLAM) enable hierarchical feature refinement by emphasizing diagnostically relevant image regions while maintaining computational efficiency. Among the proposed architectures, Res-LLANet achieved the highest overall diagnostic performance, whereas Res-BLANet provided the most computationally efficient solution, demonstrating that lightweight attention mechanisms can effectively balance diagnostic accuracy and inference speed.

In addition to improving classification performance over conventional convolutional neural network architectures trained under identical conditions, the proposed framework provides qualitative model interpretability through hierarchical attention maps that highlight image regions contributing to the classification decision. These findings suggest that lightweight hierarchical attention mechanisms can enhance discriminative feature representation while maintaining practical computational requirements, making the proposed framework a promising approach for AI-assisted parathyroid adenoma detection and a potential foundation for broader applications in oncologic medical imaging.

Despite these encouraging results, the present study has several limitations, including the relatively small independent patient cohort, single-center data acquisition, and slice-level classification design. Consequently, the reported patient-level findings should be considered preliminary and require validation using substantially larger independent multicenter datasets. Future work will focus on extending the proposed framework to volumetric multi-phase 4D-CT analysis, quantitative evaluation of attention-based explainability, lesion localization and segmentation, and comprehensive patient-level diagnostic decision support. Overall, this work demonstrates the potential of lightweight hierarchical attention mechanisms for developing computationally efficient, qualitatively interpretable, and clinically applicable AI-assisted imaging systems for parathyroid adenoma detection.

## Figures and Tables

**Table 1:** Overview of AI/ML Studies in Parathyroid Adenoma Detection.

Author	Year	Dataset	AI Model	Imaging Modality	Performance Metrics
Greene et al. [19]	2024	99 patients	Bi-LSTM, SVM	4D-CT	Bi-LSTM Acc: 92%; SVM Acc: 88%
Yeh et al. [44]	2024	458 patients	Random Forest	4D-CT + SPECT/CT	Accuracy: 90–91%
Samaras et al. [45]	2024	134 patients	LightGBM	Clinical Data	Acc: 86.9% (Adenoma)
Apostolopoulos et al. [10]	2023	648 patients	Paranet+	Scintigraphy	Acc: 98.6%; Sens: 87.3%
Apostolopoulos et al. [11]	2023	648 patients	Vision Transformer	Scintigraphy	Sens: 93.85%; Spec: 73.33%
Apostolopoulos et al. [13]	2022	632 patients	VGG19	Scintigraphy	Acc: 94.8%; Sens: 93.8%
Apostolopoulos et al. [12]	2022	100 patients	ParaNet	Scintigraphy	Acc: 96.56%; Sens: 96.38%
Yoshida et al. [40]	2022	237 patients	DL Model	Scintigraphy	Sens: 83%; mFPI: 0.31
Sandqvist et al. [17]	2022	349 patients	ML Classifier	SPECT/CT	Accuracy: 90%
Avci et al. [42]	2022	906 images	Visual DL Algorithm	NIR Autofluorescence	AUROC: 0.90; Precision: 89%
Jarabek et al. [43]	2022	93 patients	Resnet10	FCH-PET	Detection Acc: 83%
Akbulut et al. [41]	2021	50 patients	Decision Tree	NIR Autofluorescence	Accuracy: 95%

**Table 2:** Patient and slice distribution before and after data augmentation.

Dataset	Positive	Negative	Patients	Original Slices	Augmented Slices	Total Slices Used
Training	34	20	54	10,800	10,800	21,600
Validation	3	3	6	1200	0	1200
Independent Test	2	1	3	300	0	300
Overall Dataset	39	24	63	12,300	10,800	23,100

**Table 3:** Manual hyperparameter search space and selected values.

Hyperparameter	Search Space	Selected
Learning Rate	$\left\{1\times 10^{-3},5\times 10^{-4},1\times 10^{-4},1\times 10^{-5}\right\}$	$1\times 10^{-4}$
Dropout	{0.02,0.03,0.04,0.05}	0.04
L2 Regularization	$\left\{1\times 10^{-3},1\times 10^{-4},1\times 10^{-5}\right\}$	$1\times 10^{-4}$

**Table 4:** Final training configuration used for all evaluated models.

Hyperparameter	Configuration
Input Image Size	224×224
Batch Size	8
Attention Heads	8
Random Seed	42
Optimizer	Adam
Learning Rate	$1\times 10^{-4}$
Loss Function	Binary Cross-Entropy
Number of Epochs	50
Dropout Rate	0.04
L2 Weight Regularization	$1\times 10^{-4}$
Weight Initialization	Random initialization
Early stopping	No
Data Augmentation	Horizontal flip, rotation, brightness/contrast adjustment, Gaussian blur
Normalization	HU clipping [−200,300] followed by Min–Max scaling to [0,1]
Validation Strategy	10-fold cross-validation
Model Selection Criterion	Highest validation F1-score
Hyperparameter Optimization	Manual grid search (training data only)
Hardware	Intel Core i9-14900K, 128 GB RAM, NVIDIA RTX 3090 Ti (24 GB)
Framework	TensorFlow 2.14 with Keras 3

**Table 5:** 10-fold Cross Validation Results for All Models.

Model	Fold	TP	TN	FP	FN	Accuracy	Precision	Sensitivity	F1
ResNet50	1	392	512	88	208	0.753	0.817	0.653	0.726
	2	408	528	72	192	0.780	0.850	0.680	0.756
	3	512	392	208	88	0.753	0.711	0.853	0.776
	4	560	300	300	40	0.717	0.651	0.933	0.767
	5	468	520	80	132	0.823	0.854	0.780	0.815
	6	516	456	144	84	0.810	0.782	0.860	0.819
	7	452	532	68	148	0.820	0.869	0.753	0.807
	8	404	576	24	196	0.817	0.944	0.673	0.786
	9	356	568	32	244	0.770	0.918	0.593	0.721
	10	324	556	44	276	0.733	0.880	0.540	0.669
	Avg	439.2	494.0	106.0	160.8	0.778	0.828	0.732	0.764
Res-BLANet	1	576	356	244	24	0.777	0.702	0.960	0.811
	2	484	524	76	116	0.840	0.864	0.807	0.834
	3	564	452	148	36	0.847	0.792	0.940	0.860
	4	392	560	40	208	0.793	0.907	0.653	0.760
	5	568	440	160	32	0.840	0.780	0.947	0.855
	6	396	560	40	204	0.797	0.908	0.660	0.764
	7	448	516	84	152	0.803	0.842	0.747	0.792
	8	420	576	24	180	0.830	0.946	0.700	0.805
	9	436	580	20	164	0.847	0.956	0.727	0.826
	10	464	588	12	136	0.877	0.975	0.773	0.860
	Avg	474.8	515.2	84.8	125.2	0.825	0.867	0.791	0.817
Res-SLANet	1	472	504	96	128	0.813	0.831	0.787	0.808
	2	564	440	160	36	0.837	0.779	0.940	0.852
	3	444	592	8	156	0.863	0.982	0.740	0.844
	4	524	560	40	76	0.903	0.929	0.873	0.900
	5	476	572	28	124	0.873	0.944	0.793	0.862
	6	492	568	32	108	0.883	0.939	0.820	0.875
	7	492	592	8	108	0.903	0.984	0.820	0.895
	8	476	588	12	124	0.887	0.975	0.793	0.875
	9	496	576	24	104	0.893	0.954	0.827	0.886
	10	488	568	32	112	0.880	0.938	0.813	0.871
	Avg	492.4	556.0	44.0	107.6	0.874	0.926	0.821	0.867
Res-LLANet	1	512	508	92	88	0.850	0.848	0.853	0.850
	2	488	560	40	112	0.873	0.924	0.813	0.865
	3	576	480	120	24	0.880	0.828	0.960	0.889
	4	520	536	64	80	0.880	0.890	0.867	0.878
	5	480	552	48	120	0.860	0.909	0.800	0.851
	6	528	536	64	72	0.887	0.892	0.880	0.886
	7	536	516	84	64	0.877	0.865	0.893	0.879
	8	592	448	152	8	0.867	0.796	0.987	0.881
	9	476	588	12	124	0.887	0.975	0.793	0.875
	10	496	532	68	104	0.857	0.879	0.827	0.852
	Avg	520.4	525.6	74.4	79.6	0.872	0.881	0.867	0.871

**Table 6:** Performance comparison of the proposed models on the test set. Values are reported with 95% confidence intervals (CI).

Model	TP	TN	FP	FN	Accuracy	Precision	Sensitivity	F1-Score	AUC	AP
ResNet50	121	116	34	29	0.790 (0.74–0.83)	0.780 (0.70–0.83)	0.806 (0.73–0.86)	0.793 (0.74–0.84)	0.85 (0.84–0.92)	0.85 (0.84–0.93)
Res-BLANet	112	129	21	38	0.803 (0.76–0.84)	0.842 (0.77–0.89)	0.746 (0.67–0.81)	0.791 (0.73–0.85)	0.90 (0.87–0.93)	0.92 (0.88–0.94)
Res-SLANet	121	120	30	29	0.803 (0.76–0.84)	0.801 (0.73–0.86)	0.806 (0.74–0.86)	0.803 (0.75–0.86)	0.90 (0.85–0.93)	0.92 (0.89–0.95)
Res-LLANet	144	103	47	6	0.823 (0.78–0.86)	0.753 (0.69–0.81)	0.960 (0.92–0.98)	0.844 (0.80–0.88)	0.92 (0.89–0.94)	0.93 (0.90–0.94)

**Table 7:** Patient-level predictions for the test split obtained by aggregating slice-level probabilities.

Patient ID	True Label	Mean Predicted Probability	Predicted Diagnosis	Adenoma Slices	No Adenoma Slices	Total Slices
Patient 1	Adenoma	0.56381	Adenoma	56	44	100
Patient 2	Adenoma	0.4649	No Adenoma	48	52	100
Patient 3	No Adenoma	0.9553	No Adenoma	0	100	100

**Table 8:** Patient-level diagnostic performance of Res-LLANet on the independent test split (n=3 patients).

TP	TN	FP	FN	Accuracy	Precision	Sensitivity	Specificity	F1-Score
1	1	0	1	66.7% (95% CI: 9.4–99.2)	100.0% (95% CI: 2.5–100)	50.0% (95% CI: 1.3–98.7)	100.0% (95% CI: 2.5–100)	66.7% (95% CI: 9–99)

**Table 9:** Comparison of model complexity and computational cost.

Model	Trainable	Non-Trainable	Size (MB)	Mem (MB)	Test/Image (s)	FPS	Train (s/epoch)	GPU (MB)
ResNet50	34M	7936	118.15	286.30	0.1241	8.06	38.58	21,182
Res-BLANet	48M	69,504	186.05	372.10	0.0409	24.44	13.78	21,178
Res-SLANet	47M	61,312	182.07	364.15	0.0622	16.08	24.42	21,182
Res-LLANet	41M	55,168	157.00	314.01	0.1124	8.90	49.00	21,191

**Table 10:** Descriptive statistics of the fold-wise F1-scores obtained from the 10-fold cross-validation.

Model	Count	Mean	Std	Min	25%	Median	75%	Max
ResNet50	10	0.7642	0.0477	0.6694	0.7333	0.7714	0.8019	0.8190
Res-BLANet	10	0.8169	0.0375	0.7597	0.7948	0.8185	0.8502	0.8625
Res-SLANet	10	0.8669	0.0271	0.8082	0.8546	0.8732	0.8831	0.9003
Res-LLANet	10	0.8707	0.0148	0.8505	0.8555	0.8767	0.8804	0.8889

**Table 11:** Overall comparison of fold-wise F1-scores using the Friedman test.

Test	Statistic ($\chi^{2}$)	*p*-Value	Decision
Friedman Test	17.280	0.000619	Reject $H_{0}$

**Table 12:** Pairwise comparison of fold-wise F1-scores using the Wilcoxon signed-rank test with Holm–Bonferroni correction.

Comparison	Statistic	Raw *p*-Value	Adjusted *p*-Value	Significant
Res-BLANet vs. ResNet50	8.0	0.004883	0.009766	Yes
Res-SLANet vs. ResNet50	0.0	0.001953	0.011719	Yes
Res-SLANet vs. Res-BLANet	5.0	0.001953	0.005859	Yes
Res-LLANet vs. ResNet50	0.0	0.001953	0.011719	Yes
Res-LLANet vs. Res-BLANet	3.0	0.009766	0.039062	Yes
Res-LLANet vs. Res-SLANet	27.0	0.005391	0.005391	Yes

**Table 13:** Component-wise architectural comparison used for the ablation study. ✓: included, ×: excluded.

Model	BLAM	SLAM	HBA	HIA	Attention-Residual Fusion	Accuracy (%)	F1-Score (%)
ResNet50	×	×	×	×	×	79	79
Res-BLANet	✓	×	✓	✓	✓	80	79
Res-SLANet	×	✓	×	×	✓	80	80
Res-LLANet	✓	✓	✓	✓	✓	82	84

**Table 14:** Comparison of the proposed architectures with representative CNN architectures trained from scratch using identical experimental settings.

Model	Accuracy (%)	Precision (%)	Sensitivity (%)	F1-Score (%)
ConvNeXt-Base	68.20	67.80	68.60	68.19
DenseNet201	70.10	69.80	70.50	70.15
EfficientNetV2L	71.40	71.00	71.90	71.45
VGG19	63.80	63.30	64.20	63.75
InceptionV3	60.00	60.14	59.33	59.73
Xception	77.33	79.29	74.00	76.55
ResNet50	79.0	78.0	80.6	79.3
Res-BLANet	80.3	84.2	74.6	79.1
Res-SLANet	80.3	80.1	80.6	80.3
Res-LLANet	82.3	75.3	96.00	84.4

## Data Availability

The dataset used in this work is not openly available due to the research policies of Kocaeli University Hospital but is available from the corresponding author upon reasonable request and subject to institutional approval. All figures and training procedures supporting the findings of this study are included in the manuscript.
